# Mutation of *SPOTTED LEAF3* (*SPL3*) impairs abscisic acid-responsive signalling and delays leaf senescence in rice

**DOI:** 10.1093/jxb/erv401

**Published:** 2015-08-14

**Authors:** Seung-Hyun Wang, Jung-Hyun Lim, Sang-Sook Kim, Sung-Hwan Cho, Soo-Cheul Yoo, Hee-Jong Koh, Yasuhito Sakuraba, Nam-Chon Paek

**Affiliations:** ^1^Department of Plant Science, Plant Genomics and Breeding Institute, and Research Institute of Agriculture and Life Sciences, Seoul National University, Seoul 151–921, Korea; ^2^Department of Plant Life and Environmental Science, Hankyong National University, Ansung 456-749, Korea; ^3^Crop Biotechnology Institute, GreenBio Science and Technology, Seoul National University, Pyeongchang 232-916, Korea

**Keywords:** Abscisic acid, catalase activity, lesion mimic mutant, MAPKKK, reactive oxygen species, rice, *spotted leaf3*.

## Abstract

Lesion mimic mutants commonly display spontaneous cell death in pre-senescent green leaves under normal conditions, without pathogen attack. Despite molecular and phenotypic characterization of several lesion mimic mutants, the mechanisms of the spontaneous formation of cell death lesions remain largely unknown. Here, the rice lesion mimic mutant *spotted leaf3* (*spl3*) was examined. When grown under a light/dark cycle, the *spl3* mutant appeared similar to wild-type at early developmental stages, but lesions gradually appeared in the mature leaves close to heading stage. By contrast, in *spl3* mutants grown under continuous light, severe cell death lesions formed in developing leaves, even at the seedling stage. Histochemical analysis showed that hydrogen peroxide accumulated in the mutant, likely causing the cell death phenotype. By map-based cloning and complementation, it was shown that a 1-bp deletion in the first exon of *Oryza sativa Mitogen-Activated Protein Kinase Kinase Kinase1* (*OsMAPKKK1*)/*OsEDR1/OsACDR1* causes the *spl3* mutant phenotype. The *spl3* mutant was found to be insensitive to abscisic acid (ABA), showing normal root growth in ABA-containing media and delayed leaf yellowing during dark-induced and natural senescence. Expression of ABA signalling-associated genes was also less responsive to ABA treatment in the mutant. Furthermore, the *spl3* mutant had lower transcript levels and activities of catalases, which scavenge hydrogen peroxide, probably due to impairment of ABA-responsive signalling. Finally, a possible molecular mechanism of lesion formation in the mature leaves of *spl3* mutant is discussed.

## Highlight

Among the lesion mimic mutants in rice, the *spotted leaf3* (*spl3*) locus was identified by map-based cloning that encodes OsMAPKKK1. SPL3 was found to be involved in ABA-responsive signalling and to promote leaf yellowing during senescence.

## Introduction

Lesion mimic mutants display cell death in normal conditions and have a common phenotype similar to the pathogen infection-induced hypersensitive response. The study of lesion mimic mutants has provided insights on the mechanisms of programmed cell death. Lesion mimic mutants have been isolated and characterized in many plants, including maize (Hoisington *et al.*, 1982), barley (Wolter *et al.*, 1993), *Arabidopsis* (Noutoshi *et al.*, 2006), and rice (Wu *et al.*, 2008). Previous studies revealed that lesion mimic mutant genes encode distinct functional proteins such as a heat stress transcription factor (Yamanouchi *et al.*, 2002), membrane-associated proteins (Lorrain *et al.*, 2004; Noutoshi *et al.*, 2006), an ion channel family member (Balague *et al.*, 2003; Rostoks *et al.*, 2006; Mosher *et al.*, 2010), zinc finger proteins (Dietrich *et al.*, 1997; Wang *et al.*, 2005), an E3 ubiquitin ligase (Zeng *et al.*, 2004), a clathrin-associated adaptor protein (Qiao *et al.*, 2010), and splicing factor 3b subunit 3 (Chen *et al.*, 2012). Thus, the molecular mechanisms of lesion formation seem to be very complicated in plants. Over-accumulation of reactive oxygen species (ROS), such as superoxide radical (O_2_^−^) and hydrogen peroxide (H_2_O_2_), is closely associated with lesion formation, as confirmed in several lesion mimic mutants (Qiao *et al.*, 2010; Shirsekar *et al.*, 2014). It is also reported that lesion mimic phenotypes are affected by the light-intensity and light/dark diurnal cycle (Kusumi *et al.*, 2000). Thus, endogenous signalling pathways to external stress stimuli are likely impaired in several lesion mimic mutants.

The highly conserved mitogen-activated protein kinase (MAPK) cascade functions in the response to external environmental stimuli, acting in the transduction of extracellular cues to intercellular targets (Widmann *et al.*, 1999). The MAPK cascade comprises MAPKs, MAPK kinases (MAPKKs), and MAPKK kinases (MAPKKKs) (Schaeffer and Weber, 1999; Widmann *et al.*, 1999; Ligterink, 2000). The *Arabidopsis thaliana* genome has 20 MAPKs, 10 MAPKKs, and more than 80 MAPKKKs (Colcombet and Hirt, 2008). By contrast, the rice genome encodes 75 MAPKKKs, 8 MAPKKs, and 17 MAPKs (Agrawal *et al.*, 2003, Hamel *et al.*, 2006, Rao *et al.*, 2010). *Arabidopsis* and rice have many more MAPKKKs than MAPKs or MAPKKs, leading to complicated and variable regulatory cascades. Several *Arabidopsis* MAPKKKs have been characterized, and they regulate various biological processes, such as cytokinesis (Takahashi *et al.*, 2010), stomatal development (Kim *et al.*, 2012), and the responses to biotic (Kieber *et al.*, 1993; Frye *et al.*, 2001) and abiotic stresses (Gao and Xiang, 2008; Huang *et al.*, 2014). However, studies of MAPKKKs in rice remain limited.

Phytohormone signalling pathways also have important roles in responding to external stress stimuli. Jasmonic acid (JA)-, salicylic acid (SA)-, and ethylene-responsive signalling pathways are tightly associated with the resistance to biotrophic and necrotrophic pathogens (Robert-Seilaniantz *et al.*, 2007), and are important for the response to abiotic stresses (Clarke *et al.*, 2009; Brossa *et al.*, 2011; Lei *et al.*, 2011). Abscisic acid (ABA) positively regulates the response to several abiotic stresses, e.g. drought and osmotic stresses, by promoting the closure of stomata (Radin, 1984; Fujita *et al.*, 2005). *Arabidopsis* transgenic plants overexpressing several ABA-induced bZIP transcription factors, such as ABA-responsive element (ABRE) binding protein (AREB) and ABRE binding factor (ABF), exhibited tolerance to drought and/or osmotic stresses (Fujita *et al.*, 2005, 2009; Yoshida *et al.*, 2010). In addition to abiotic stress response, ABA also controls various developmental processes, including seed germination, root elongation, leaf senescence, and seed development (De Smet *et al.*, 2006; Nakashima *et al.*, 2013). These phytohormone signalling pathways are, at least in part, regulated by specific MAPK cascades. One of the *Arabidopsis* MAPKKKs, *CONSTITUTIVE TRIPLE RESPONSE1* (*CTR1*) modulates ethylene signalling by promoting *ETHYLENE INSENSITIVE3* (*EIN3*) transcription, together with MAPKK9-MAPK3/MAPK6 (Yoo and Sheen, 2008). Another *Arabidopsis* MAPKKK, *ENHANCED DISEASE RESISTANCE1* (*EDR1*), negatively regulates the SA-dependent defence pathway. The *edr1* knockout mutant showed resistance to powdery mildew disease caused by *Erysiphe cichoracearum*, but SA-deficient or SA signalling-related mutants completely suppressed this phenotype (Frye *et al.*, 2001). However, the relationship between phytohormone signalling and MAPK pathways largely remains unknown.

In this study, the rice *spl3* mutant, which produces spontaneous cell death lesions on its leaf blades and shows excessive accumulation of H_2_O_2_, was analysed. Map-based cloning showed that the *spl3* locus encodes a putative kinase protein, OsMAPKKK1. The *spl3* mutant is strongly insensitive to ABA treatment and delays leaf senescence, probably due to reduced expression of ABA signalling-related genes. In the *spl3* leaves, a significant decrease of catalase activity, which functions in scavenging H_2_O_2_ in the cells, was found. These *spl3* data provide insights into the molecular function of *SPL3* in ABA-responsive signalling in plants.

## Materials and methods

### Plant materials and growth conditions

The *spl3* mutant was originally generated by γ-ray irradiation of a Japanese *japonica* rice cultivar ‘Norin8’ (Yoshimura *et al.*, 1997). The wild-type Norin8 and *spl3* mutant were grown in the paddy field (natural long day conditions at 37° N latitude, Suwon, Korea) or in the growth chambers. The chamber experiments were performed under short day (SD) conditions [10-h light with normal intensity (300 μmol m^−2^ sec^−1^) at 30 °C and 14-h dark at 20 °C], or continuous light with 30 °C for 10h and 20 °C for 14h. For phenotypic characterization and map-based cloning of *spl3* mutant, all the plants were grown in the paddy field.

### Detection of ROS

The detection of ROS accumulation was conducted as previously described (Sakuraba *et al.*, 2013). To determine hydrogen peroxide (H_2_O_2_) and superoxide anion (O_2_^−^), leaf samples of 1-month-old plants grown in the growth chamber under SD or continuous light conditions were transferred in DAB staining solution containing 0.1% 3,3′-diaminobenzidine or in nitroblue tetrazolium (NBT) staining solution including 0.05% nitroblue tetrazolium chloride in 50mM sodium phosphate buffer, and incubated for 6h with gentle shaking. After staining, chlorophyll was completely removed by incubation with 90% ethanol at 80 °C. H_2_O_2_ accumulation was also measured using the Amplex Red Hydrogen Peroxide/Peroxidase Assay kit (Life Technologies, USA) according to the manufacturer’s protocol.

### Genetic analysis and map-based cloning

For genetic analysis, an F_2_ population was developed from crossing between a *japonica*-type *spl3* mutant and Korean Tongil-type cultivar Milyang23, which was derived from hybridization of *indica* × *japonica* rice cultivars. The *spl3* locus was previously mapped to the short arm of chromosome 3 (Yoshimura *et al.*, 1997). In this study, a mapping population of 1800 F_2_ individuals from the cross between *spl3* and Milyang23 was used for locating and fine mapping of the *spl3* locus. Genomic DNA was extracted from young leaves of each F_2_ individual line. The newly designed markers using Milyang23 sequence data (Lim *et al.*, 2014) were used to narrow down the genomic region of *spl3* locus on chromosome 3; these markers included sequence-tagged-site (STS) markers (Supplementary Table S1 at *JXB* online).

### Stress treatments

Stress treatments were performed as described (Kim *et al.*, 2003). Two-week-old wild-type (WT) seedlings (cv. Norin8) were treated with dehydration, NaCl (150mM), mannitol (500mM), SA (100 μM), methyl jasmonate [MeJA (100 μM)], 1-aminocyclo-propane-1-carboxylic acid [ACC (10mM)], and different concentrations of ABA (5–50 μM). To check the senescence phenotype of WT and *spl3* leaves under treatments of four senescence-promoting phytohormones (ABA, ACC, SA, and MeJA), detached leaf discs from 1-month-old plants were floated on the 3mM MES (pH 5.8) buffer supplemented with 50 μM ABA, 10mM ACC, 100 μM SA, and 100 μM MeJA and incubated for 4 d under continuous light conditions. To check the phenotype under drought and osmotic stresses, WT and *sp*l3 plants were grown under LD conditions for 2 month. For drought stress treatment, plants were dehydrated for 5 d at 25 °C and 50% humidity, and then rehydrated again for investigating the recovery of wilting phenotype. For the salt stress assay, plants were transferred to 500mM mannitol and incubated for 5 d.

### Chlorophyll measurement

For the measurement of total chlorophyll (Chl) concentration, photosynthetic pigments were extracted from the leaf tissues with 80% ice-cold acetone. Chl concentrations were determined by spectrophotometry as described previously (Porra *et al.*, 1989).

### SDS-PAGE and immunoblot analysis

Total protein extracts were prepared from the leaf tissues. To extract total proteins, leaf tissues of rice grown in the paddy field were ground in liquid nitrogen and 10mg aliquots were homogenized with 100 μl of sample buffer (50mM Tris, pH 6.8; 2mM EDTA; 10% glycerol; 2% SDS; and 6% 2-mercaptoethanol). Homogenates were centrifuged at 10 000 ×*g* for 3min, and supernatants were denatured at 80 °C for 5min. Four microlitres of each sample was subjected to 12% (w/v) SDS-PAGE and resolved proteins were electroblotted onto a Hybond-P membrane (GE Healthcare, USA). Antibodies against the photosystem proteins Lhcb1, Lhcb2, Lhcb4, Lhca1, Lhca2, and D1 (Agrisera, Sweden) were used for immunoblot analysis. The level of each protein was examined using the ECL system (WESTSAVE kit, AbFRONTIER, Korea) according to the manufacturer’s protocol.

### Measurement of ion leakage rates

Ion leakage rates were measured as described previously (Lee *et al.*, 2015). Briefly, membrane leakage was determined by the amount of electrolytes (or ions) leaking from rice leaf discs (1cm^2^). Three leaf discs from each treatment were immersed in 6ml of 0.4M mannitol at room temperature with gentle shaking for 3h, and initial conductivity of the solution was measured with a conductivity meter (CON 6 METER, LaMotte Co., USA). Total conductivity was determined after sample incubation at 85 °C for 20min. Ion leakage rate is expressed as the percentage of initial conductivity divided by the total conductivity.

### Stomatal aperture analysis

The stomatal aperture of abaxial leaf epidermal strips was analysed as previously described (Xing *et al.* 2007) with minor modifications. Leaf discs from 3-week-old plants grown under SD conditions were incubated in 3mM MES buffer (pH 6.15) containing 50mM KCl (MES-KCl) for 2h under light (22 °C) to open stomata. Leaf discs were then transferred to the MES-KCl buffer containing 5 μM ABA for 4h. Stomatal cells were observed by Field-Emission Scanning Electron Microscopy (AURIGA, Carl ZEISS, Germany).

### Complementation test

For complementation of the *spl3* mutation, a full-length cDNA of *SPL3* was ligated into the pMDC32 Gateway binary vector containing the *35S* promoter (Curtis and Grossniklaus, 2003). The *35S:SPL3* construct in the pMDC32 plasmid was introduced into the calli generated from the mature embryos of *spl3* mutant seeds by Agrobacterium (strain LBA4404)-mediated transformation (Jeon *et al.*, 2000). Transformants were confirmed by PCR using the specific primers listed in Supplementary Table S1 at *JXB* online.

### Reverse transcription-quantitative real-time PCR analysis

Total RNA was extracted from the 4-week-old plants with the MG Total RNA Extraction Kit (Macrogen, Korea), including DNase I treatment step for removing the possible contamination of genomic DNAs. First-strand cDNAs were synthesized with 2 μg of total RNA in a 25 µl volume using M-MLV reverse transcriptase and oligo(dT)_15_ primer. The 20 µl of reverse transcription (RT)-quantitative real-time PCR (qPCR) mixture contained 2 µl of the RT mixture, 10 µl of 2X GoTar PCR mix (Roche), and 0.25 µl of the primers. The qPCR was performed on the Light Cycler 2.0 (Roche Diagnostics, Germany). The qPCR conditions were 95 °C for 2min, followed by 45 cycles at 95 °C for 5 s, 59 °C for 15 s, and 72 °C for 10 s. The relative expression of each gene was calculated using the 2^−∆∆C^_T_ methods (Livak and Schmittgen, 2001). The primers used for qPCR are listed in Supplementary Table S1 at *JXB* online.

### Catalase assay

Catalase activity in the rice leaves was analysed using the Sigma Catalase Assay kit (Sigma-Aldrich, USA) following the manufacturer’s instructions.

## Results

### Phenotypic characterization of the *spl3* mutant

**Fig. 1.**
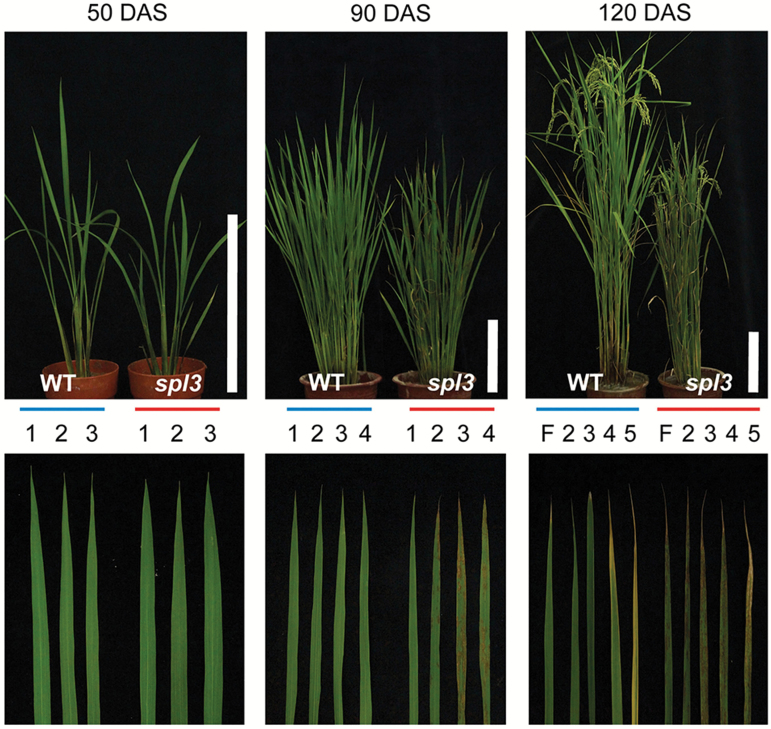
Phenotypic characterization of the *spl3* mutant. Whole plants (upper panels) and leaf blades (lower panels) of 50-, 80-, and 120-DAS WT and *spl3* mutant grown in the paddy field. F, flag leaf; 1 to 5, 1st to 5th leaf blade from the top. Scale bar, 30cm.

A single recessive *spl3* mutant in rice was isolated from the M2 population of *japonica* cultivar ‘Norin8’ irradiated with gamma rays (Yoshimura *et al.*, 1997). First, the *spl3* phenotype was examined in the paddy field. At early tillering stage [50 d after seeding (DAS)], the phenotype of *spl3* mutant appeared to be quite similar to that of the WT, without any lesion development (Fig. 1, left panels). At late-tillering stage (90 DAS), lesions gradually appeared, mainly in the old leaves and from the tip region (Supplementary Fig. S1 at *JXB* online), with only a few in young leaves (Fig. 1, middle panels). At heading stage (120 DAS), lesion formation became more severe and finally the dark-brown spots expanded to all leaves including the flag leaf (Fig. 1, right panel). Interestingly, the growth of *spl3* mutant (plant height) became clearly retarded as the lesion mimic phenotype appeared (Fig. 1; Supplementary Fig. S2 at *JXB* online), which led to a decrease of several agronomic traits, especially spikelet fertility and panicle length (Supplementary Fig. S3 at *JXB* online). These results indicate that lesion formation in *spl3* mutant is closely related with the leaf age, between late-tillering and heading stages.

### Excessive accumulation of ROS causes development of cell death lesions in leaf blades

**Fig. 2.**
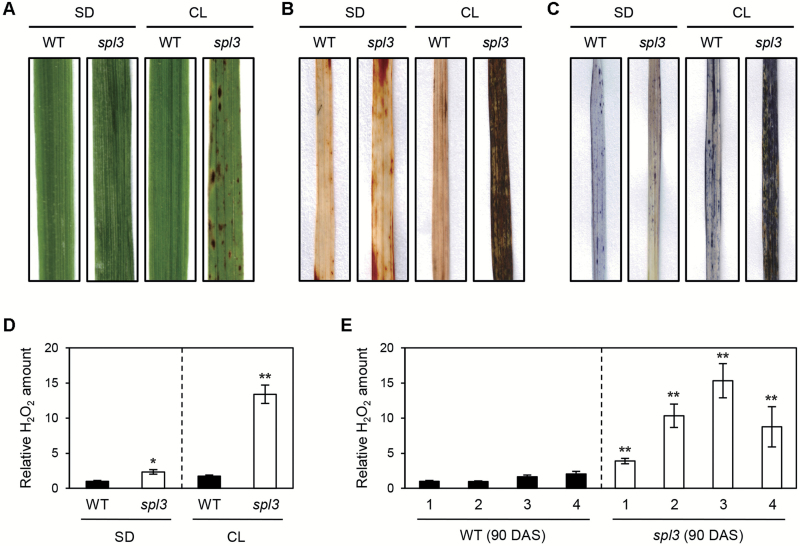
ROS accumulation in the *spl3* leaf blades. (A) WT and *spl3* mutant were grown under SD or CL conditions at 30 °C for 1 month in the growth chamber. (B, C) The accumulation of H_2_O_2_)and O_2_^−^ were detected by staining with 3,3′-diaminobenzidine (DAB) (B) and NBT (C), respectively. (D, E) H_2_O_2_ quantification was performed using Amplex Red. 2nd leaves from 30-DAS WT and *spl3* mutant grown under SD and CL conditions (D), and 1st to 4th leaves from the main culm of 90-DAS WT and *spl3* mutant grown in the paddy field (E). Mean and standard deviation values were obtained from more than three biological replicates. These experiments were repeated three times with similar results. (D, E) Statistical analysis using Student’s *t*-test, *P<0.05; **P< 0.01.

To examine the effect of the diurnal light-dark cycle on lesion formation in the *spl3* mutant, WT (cv. Norin8) and *spl3* mutant were grown in the growth chamber (300 μmol m^−2^ s^−1^) under SD; 10-h light, 30 °C/14-h dark, 20 °C) or continuous light (CL). Under SD, the *spl3* mutant developed no lesions on the leaves even after 30 DAS (Fig. 2A). Under CL conditions, however, reddish spots with some necrotic lesions appeared in the *spl3* leaves (Fig. 2A).

Excessive accumulation of ROS causes leaf variegation and/or necrotic lesions in some variegated-leaf mutants in rice (Li *et al.*, 2010; Han *et al.*, 2012; Sakuraba *et al.*, 2013). Thus, the levels of two kinds of ROS, O_2_^−^ and H_2_O_2_, were examined in the *spl3* leaves grown under SD or CL conditions, using two staining methods: NBT for O_2_^−^ and DAB for H_2_O_2_. Under SD, the O_2_^−^ and H_2_O_2_ levels in the *spl3* leaves were almost the same as those of the WT. However, O_2_^−^ and H_2_O_2_ accumulated in the *spl3* leaves under CL compared with the WT leaves (Fig. 2B, C). H_2_O_2_ production under SD and CL conditions was also confirmed by quantification using the hydrogen peroxide/peroxidase assay kit. Consistent with the results of DAB staining (Fig. 2B), the *spl3* leaves had significantly higher H_2_O_2_ levels under CL conditions (Fig. 2D). Furthermore, it was found that at 90 DAS, the *spl3* leaves had much higher H_2_O_2_ levels than the WT leaves, especially in the 3rd and 4th leaves, which had many lesions (Fig. 2E).

### Map-based cloning of *SPL3*

**Fig. 3.**
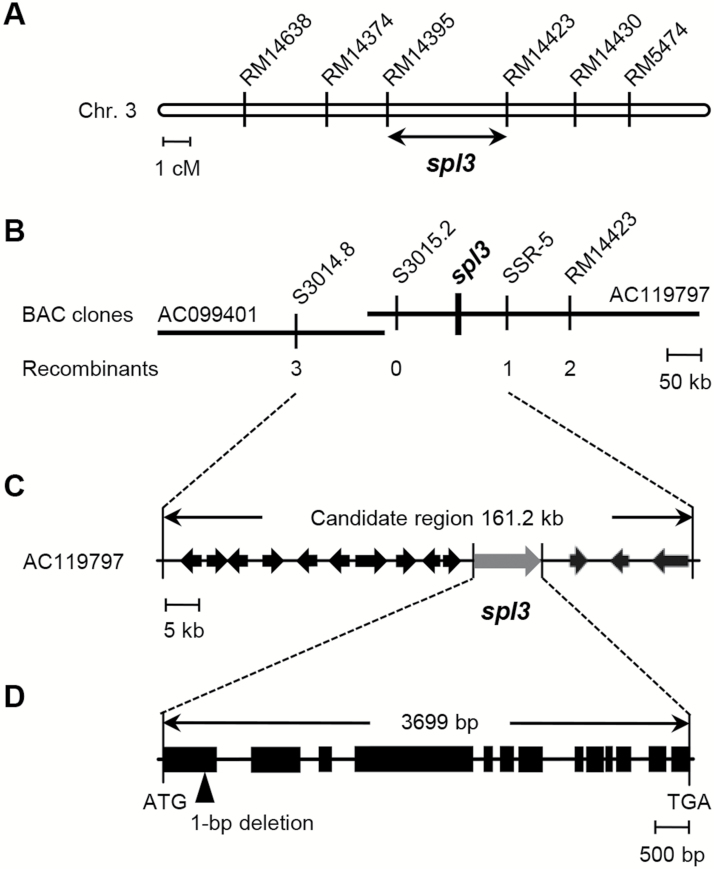
Map-based cloning of the *spl3* locus. (A) Genetic mapping of the *spl3* locus. The *spl3* locus was initially narrowed down to the region between two SSR markers, RM14395 and RM14423, on the short arm of chromosome 3. The PCR primer sequences of SSR and STS markers are listed in Supplementary Table S1 at *JXB* online. (B, C) Fine physical mapping of the *spl3* locus. The *spl3* locus region was narrowed down to a 161.2-kb interval between S3015.2 and SSR-5 markers using six recombinants in F_2_ individuals. (D) The *SPL3* gene structure. Thirteen exons and 11 introns are designated by black rectangles and lines, respectively; a 1-bp deletion occurs in the 1st exon, leading to a frameshift mutation.

To isolate the *SPL3* gene, a map-based cloning was performed using 1771 F2 plants that were generated from a cross of *spl3* (*japonica*) mutant and Milyang23 (a Tongil-type *indica/japonica* hybrid cultivar). The *spl3* locus was mapped to a 415-kb interval between RM14395 and RM14423 on chromosome 3 (Fig. 3A). Using two STS and one simple sequence repeat (SSR) markers, the *spl3* locus was further delimited to a 161.2-kb interval between S3015.2 and SSR-5, in the BAC clones AC099401 and AC119797 (GenBank accession number) (Fig. 3B). In this genomic region, 16 candidates were found based on the Rice Functional Genomic Expression Database of the Salk Institute Genomic Analysis Laboratory (http://signal.salk.edu/cgi-bin/RiceGE). Sixteen expressed genes were cloned by RT-PCR or genomic PCR. As a result, a 1-bp deletion was identified in the first exon of a candidate gene, LOC_Os03g06410 (Fig. 3C, D), resulting in a frameshift mutation (Supplementary Fig. S1 at *JXB* online) that leads to premature translational termination (Supplementary Fig. S4 at *JXB* online). LOC_Os03g06410 encodes a putative MAPKKK, which is orthologous to AtEDR1 (*Arabidopsis thaliana* Enhanced Disease Resistance1; Frye *et al.*, 2001). Based on this orthology, the locus had been named OsEDR1 (Kim *et al.*, 2003; Shen *et al.*, 2011), although the same group renamed it OsACDR1 (*Oryza sativa* Accelerated Cell Death and Resistance1) because the function of OsEDR1 was considerably different from that of AtEDR1 (Kim *et al.*, 2009*a*). It was also named OsMAPKKK1 (Rao *et al.*, 2010). SPL3/OsEDR1/OsACDR1/OsMAPKKK1 comprises 1018 amino acids with a protein kinase domain at the C-terminal region, which was abrogated in the *spl3* allele (Supplementary Fig. S3 at *JXB* online).

To confirm that the mutation in *SPL3* caused the *spl3* phenotype, a complementation test was performed. As a result, five independent transgenic lines did not show any lesion mimic phenotype at 60 DAS, when spots developed clearly on the mature leaves of *spl3* mutant (Supplementary Fig. S5 at *JXB* online). These results indicate that the 1-bp deletion in the exon 1 of *OsMAPKKK1* is responsible for the *spl3* mutation.

### The transcript accumulation of *SPL3* is dependent on leaf age

To examine SPL3 function, *SPL3* expression was examined in various tissues of 2-week-old WT plants by RT-qPCR. *SPL3* transcripts accumulated the most in the leaf sheath and leaf blade (Supplementary Fig. S6A at *JXB* online). Lesions in *spl3* mutant are predominant in the tip area of older leaf blades both in the field and in growth chamber conditions (Figs 1 and 2; Supplementary Fig. S1). Thus, *SPL3* expression was subsequently examined in two different sections (top and middle) of three different stages of leaf blades (flag, 2nd, and 3rd leaves). In all leaves, *SPL3* transcript levels were significantly lower in the top area, especially in flag leaves (Supplementary Fig. S6B). Together, these data indicate that *SPL3* has an important role in protecting young leaves from the generation of necrotic lesions.

### ABA-responsive signalling is impaired in the *spl3* mutant

*SPL3* expression was shown to be rapidly and transiently regulated by diverse environmental stresses over a short timeframe of 30–120min (Kim *et al.*, 2003). To further study the *SPL3* function under abiotic stresses, the time-course expression of *SPL3* was examined for 24h in response to three abiotic stresses (drought, mannitol, and salt) and four hormones (ABA, ethylene, SA, and JA) (Supplementary Fig. S7 at *JXB* online). *SPL3* transcript levels were found to decrease rapidly in response to ABA, SA, and MeJA, and decrease slowly in response to ethylene (ACC) and three abiotic stresses, suggesting that *SPL3* has an important role in both hormone-responsive and abiotic stress-responsive signalling. Thus, the response of *spl3* mutant to senescence-promoting hormones, including ABA, ethylene, SA, and MeJA (Kusaba *et al.*, 2013), was examined. It was found that the *spl3* mutant showed a stay-green phenotype under ABA- and ACC-induced senescence conditions, but no significant phenotypic difference under SA- and MeJA-induced senescence conditions (Supplementary Fig. S8 at *JXB* online), indicating that both ABA and ethylene signalling pathways were impaired in the *spl3* leaves.

**Fig. 4.**
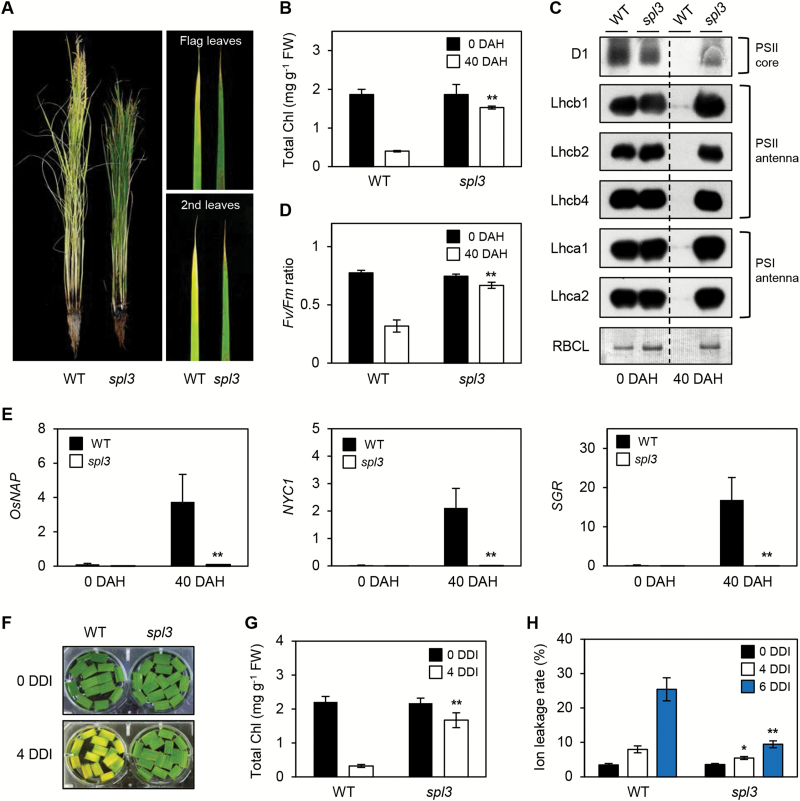
The *spl3* mutant shows a stay-green phenotype during leaf senescence. (A) Phenotype of WT and *spl3* mutant at 40 DAH. (B–D) Changes of total Chl levels (B), chloroplast protein levels (C), and *Fv/Fm* ratios (D) in WT and *spl3* mutant at 0 and 40 DAH. (E) Transcript levels of the three SAG genes in the WT and *spl3* mutant at 0 and 40 DAH were measured by RT-qPCR. Transcript levels of *OsNAP*, *NYC1*, and *SGR* were normalized to the transcript levels of *OsUBQ5*. (F–H) The *spl3* leaves stay green during dark-induced senescence. Changes of visible phenotype (F), total Chl levels (G), and ion leakage rates (H) in the WT and *spl3* mutant before and after 4 DDI were examined. (G, H) Black, white, and grey bars indicate 0, 4, and 6 DDI, respectively. Statistical analysis using Student’s *t*-test, *P<0.05; **P<0.01. Mean and standard deviation values were obtained from more than three biological replicates. These experiments were repeated twice with similar results.

Next, the senescence phenotype of *spl3* mutant in the field was checked. During the pre-senescent phase, the leaf colour of the *spl3* mutant was almost the same as that of the WT (Fig. 1). At the senescent phase [40 d after heading (DAH)], however, the *spl3* leaves exhibited a strong stay-green phenotype (Fig. 4A), even though the heading date of *spl3* mutant was the same as that of the WT (Supplementary Fig. S9 at *JXB* online). Consistent with this, Chl and photosynthesis-related proteins (D1, Lhcb1, Lhcb2, Lhcb4, Lhca1, Lhca2, and RbcL) were retained in the *spl3* leaves (Fig. 4B, C). In parallel, the *Fv/Fm* ratio, and the photosynthetic efficiency of photosystem II, were also retained in the *spl3* leaves compared with the WT (Fig. 4D). The expression of three typical senescence-associated genes (SAGs) was also investigated for Chl catabolism: *STAYGREEN* (*SGR*; Park *et al.*, 2007), *NON-YELLOW COLOURING1* (*NYC1*; Kusaba *et al.*, 2007), and *OsNAP* (Liang *et al.*, 2014). The transcript levels of these three SAGs were down-regulated in the *spl3* mutant at 40 DAS compared with the WT (Fig. 4E).

The stay-green phenotype of the *spl3* mutant was also confirmed during dark-induced senescence. After 4 d of dark incubation (4 DDI), the leaf discs of the WT turned completely yellow, while those of the *spl3* mutant remained green (Fig. 4F), with higher Chl levels (Fig. 4G) and lower ion leakage rates (Fig. 4H). Furthermore, *SPL3* expression increased during both natural and dark-induced senescence (Supplementary Fig. S10 at *JXB* online).

### ABA signalling-related phenotype of the *spl3* mutant

**Fig. 5.**
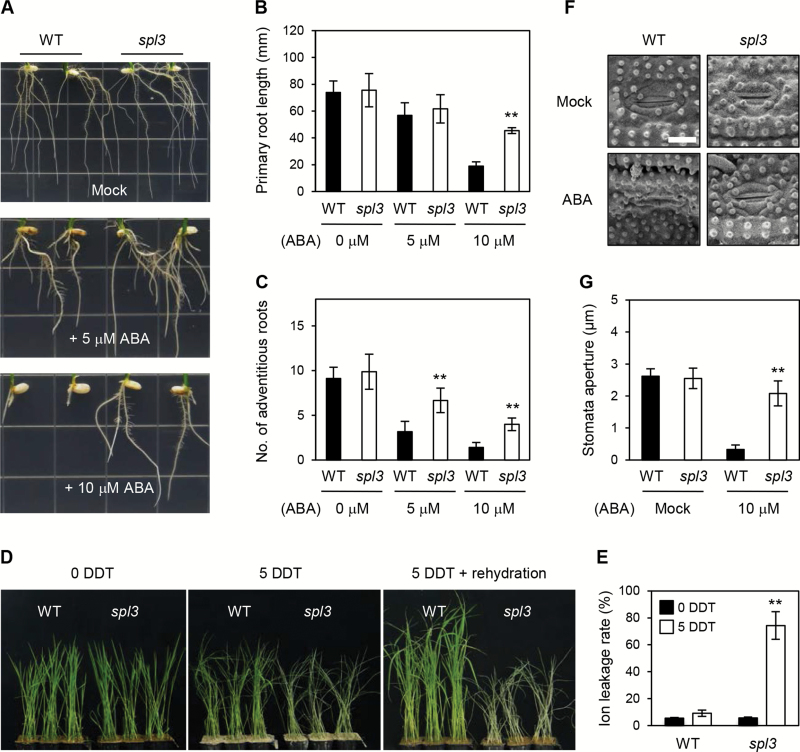
The *spl3* mutant is less responsive to exogenous ABA and hypersensitive to drought stress. (A) Effect of different concentrations of ABA (0, 5, and 10 μM) on root growth is significantly reduced in the *spl3* mutant (each square = 1.8×1.8cm^2^). (B, C) The root length (B) and number of adventitious roots (C) in 8-d-old plants were measured. (D, E) The *spl3* mutant shows a sensitive phenotype to drought stress. Four-week-old WT and *spl3* plants grown under LD conditions were dehydrated for 5 d (5 DDT). The phenotype (D) and ion leakage rate (E) before and after dehydration are shown, respectively. (F, G) Effect of ABA (10 μM) on stomatal closure is significantly reduced in the *spl3* leaves (F), and stomatal apertures were measured (G). White bar, 5 μm. Statistical analysis using Student’s *t*-test, **P<0.01). Mean and standard deviation values were obtained from more than three biological replicates. These experiments were repeated three times with similar results. DT, days of treatment. DDT, days of drought treatment.

Next, it was tested whether the *spl3* mutant shows altered phenotypes for ABA-related processes. The root phenotype of *spl3* mutant in ABA-containing media (Fig. 5A–C) was investigated. The WT showed remarkably retarded root development in the presence of ABA; however, the *spl3* mutant produced longer primary roots and more adventitious roots than the WT. Next, the phenotype of the *spl3* mutant was examined under abiotic stress conditions, such as drought and osmotic stresses. During 5 d of dehydration, the *spl3* mutant wilted much earlier than the WT and did not recover after rehydration (Fig. 5D, E). Similarly, the *spl3* mutant was more sensitive to osmotic stress (500mM mannitol) (Supplementary Fig. S11 at *JXB* online). The stomata in the *spl3* leaf surfaces was also observed, which revealed that stomatal closure in the *spl3* mutant was nearly insensitive to ABA treatment (Fig. 5F, G). In contrast to these ABA-related phenotypes, which differed between the WT and *spl3* mutant, the seed germination rate did not differ between the WT and *spl3* (Supplementary Fig. S12 at *JXB* online). Taken together, these data indicate that SPL3 is involved in some, but not all ABA-responsive pathways.

### Altered expression of ABA signalling-related genes in the *spl3* mutant

**Fig. 6.**
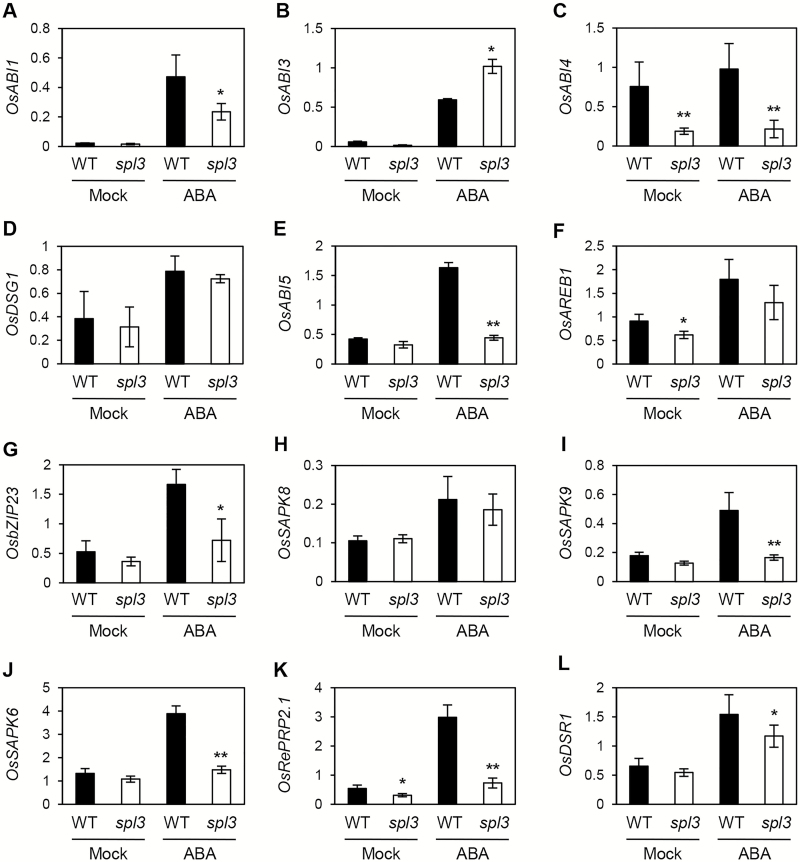
Expression of ABA signalling-related genes in the *spl3* mutant after ABA treatment. The 10-d-old WT and *spl3* seedlings were transferred to MS solution containing 10 μM ABA and were sampled after 6h for RT-qPCR analysis. RT-qPCR was used to measure the relative transcript levels of (A) *OsABI1*, (B) *OsABI3*, (C) *OsABI4*, (D) *OsDSG1*, (E) *OsABI5*, (F) *OsAREB1*, (G) *OsbZIP23*, (H) *OsSAPK8*, (I) *OsSAPK9*, (J) *OsSAPK6*, (K) *OsRePRP2.1*, and (L) *OsDSR1* and transcript levels were normalized to the transcript levels of *OsUBQ5*. Mean and standard deviation values were obtained from more than three biological replicates. These experiments were repeated twice with similar results. Statistical analysis using Student’s *t*-test, *P<0.05; **P<0.01).

Because of ABA insensitivity in root development, leaf senescence, and abiotic stresses, it was hypothesized that ABA-responsive signalling is severely compromised in the *spl3* mutant. To examine this, the expression levels of the ABA signalling-associated genes were compared between the WT and *spl3* mutant after 6h of ABA treatment. Based on previous reports of ABA signalling in rice (Zhou *et al.*, 2008; Park *et al.*, 2010; Tseng *et al.*, 2013), the ABA signalling-associated genes involved in seed germination (*OsABI1*, *OsABI3*, *OsABI4*, and *OsDSG1*), seed germination and development (*ABI5*), abiotic stress responses (*OsAREB1*, *OsbZIP23*, *OsSAPK8*, and *OsSAPK9*), and root development (*OsSAPK6*, *OsRePRP*, and *OsDSR1*) were investigated. Among them, several ABA-associated genes, including *ABI1*, *ABI4*, *ABI5*, *OsbZIP23*, and *OsSAPK9*, *OsSAPK6*, and *OsRePRP* were significantly down-regulated in the *spl3* mutant after ABA treatment (Fig. 6). Thus, it is probable that the strong repression of ABA signalling-related genes leads to the ABA insensitivity in the *spl3* mutant. By contrast, the expression of *OsABI3* (Fig. 6B) and *OsDSG1* (Fig. 6D), key regulators of seed germination in rice (Park *et al.*, 2010), were not down-regulated in the *spl3* mutant.

In parallel, ABA signalling-associated genes (*OsABI1*, *OsABI5*, *OsbZIP23*, and *OsSAPK9*) were also found to be down-regulated in the *spl3* mutant during natural senescence (Supplementary Fig. S13 at *JXB* online), suggesting that the down-regulation of these genes, in addition to *OsNAP* (Fig. 4E), is also associated with delayed senescence of *spl3* leaves. A previous study reported that the expression levels of genes related to ethylene biosynthesis were severely suppressed in the knockout mutant of *OsEDR1* (Shen *et al.*, 2011). During senescence, two ethylene-signalling-related SAGs, *ETHYLENE INSENSITIVE2* (*EIN2*) and *EIN3*, were found to be down-regulated in the *spl3* mutant, as were two ethylene biosynthetic genes, *ACC SYNTHASE1* (*ACS1*) and *ACS2* (Supplementary Fig. S14 at *JXB* online), suggesting that the impairment of both ABA- and ethylene-responsive signalling pathways results in the delayed leaf senescence phenotype of the *spl3* mutant.

### ABA insensitivity in the *spl3* mutant leads to down-regulation of catalase expression

Catalase (CAT) scavenges H_2_O_2_, and its physiological functions have been widely studied in *Arabidopsis* (Mhamdi *et al.*, 2010). In *Arabidopsis*, ABA treatment induces expression of the three *CAT* genes (Xing *et al.*, 2007). Because ABA signalling is compromised in the *spl3* mutant (Figs 4, 6), it was next tested whether gene expression and enzymatic activity of catalases are also significantly down-regulated in the *spl3* mutant.

**Fig. 7.**
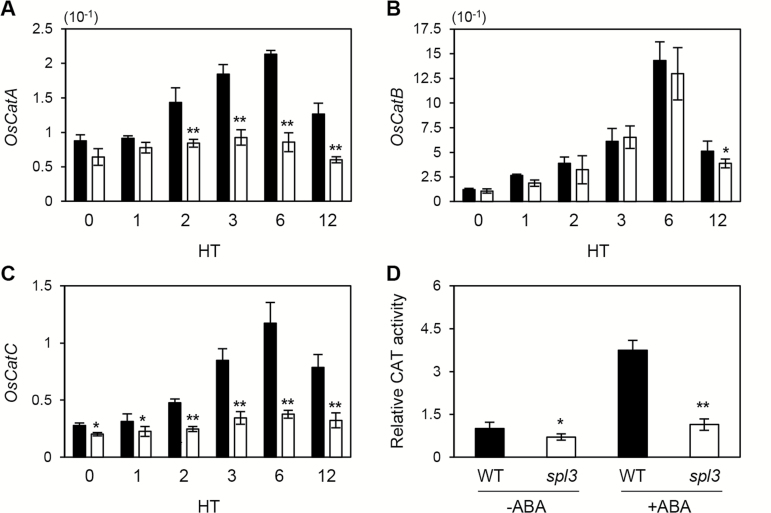
Catalase activity is reduced in the *spl3* mutant after ABA treatment. (A–C) The leaf discs were taken from the 2nd leaves of 1-month-old WT and *spl3* plants grown under LD conditions and were treated with 20 μM ABA for 12h, and then were sampled for RT-qPCR. RT-qPCR was used to measure the relative transcript levels of *OsCatA* (A), *OsCatB* (B), and *OsCatC* (C), which were normalized to the transcript levels of *OsUBQ5*. (D) Relative catalase activity in leaves from 1-month-old WT and *spl3* seedlings treated for 6h with 20 μM ABA. Mean and standard deviation values were obtained from more than three biological replicates. These experiments were repeated three times with similar results. HT, hours of treatment. Statistical analysis using Student’s *t*-test, *P<0.05; **P<0.01.

To this end, the expression levels of rice catalase genes was investigated in the *spl3* mutant. Rice has three *CAT* homologues, *OsCatA*, *OsCatB*, and *OsCatC* (Iwamoto *et al.*, 2000; Mhamdi *et al.*, 2010). The three *OsCAT* genes were found to be significantly up-regulated in the WT, in response to ABA treatment (Fig. 7A, C), similar to *Arabidopsis* catalases (Xing *et al.*, 2007). Among these three rice catalases, *OsCatA* and *OsCatC* mRNA levels were significantly down-regulated compared with the WT (Fig. 7A, C), whereas *OsCatB* mRNA levels were not altered in the *spl3* mutant after ABA treatment (Fig. 7B). Furthermore, the CAT activities of *spl3* and WT leaves were compared, and the *spl3* leaves were found to have significantly lower CAT activity than the WT (Fig. 7D).

## Discussion

By map-based cloning, the *SPL3* locus was found to encode OsMAPKKK1, a rice homologue of *Arabidopsis* EDR1 (Kim *et al.*, 2003). Thus, it was also termed OsEDR1 (Kim *et al.*, 2003; Shen *et al.*, 2011). Previous studies of OsEDR1 mainly focused on its function in the biotic stress-responsive pathway; transgenic rice plants overexpressing *OsEDR1* (*OsEDR1*-OX) displayed spontaneous hypersensitive response-like spots on the mature leaves, and concurrent up-regulation of defence-related genes and accumulation of phenolic compounds and phytoalexins. As a result, the *OsEDR1*-OX plants gained enhanced resistance to the rice blast fungal disease *Magnaporthe grisea* (Kim *et al.*, 2009*a*). Moreover, another group reported that *osedr1* knockout plants have enhanced resistance to the bacterial blight disease *Xanthomonas oryzae* pv. *oryza*. This resistance was closely associated with increased accumulation of SA and JA and thus up-regulation of SA- and JA-associated gene expression, in parallel with decreased accumulation of the direct ethylene precursor ACC and down-regulation of ethylene-related gene expression (Shen *et al.*, 2011). Finally, the authors concluded that OsEDR1 is not a functional homologue of AtEDR1 because of the different responses to pathogen attacks (Frye *et al.*, 2001; Tang *et al.*, 2005). Here it is shown that SPL3/OsMAPKKK1/OsEDR1 functions as a transducer of ABA-responsive signalling and that the *spl3* mutant showed strong ABA insensitivity, leading to several interesting phenotypes, such as delayed leaf senescence (Fig. 4; Supplementary Fig. S15 at *JXB* online).

### SPL3 regulates several ABA-responsive signalling pathways in rice

In this study, it was found that the *spl3* mutant was insensitive to ABA in several ABA-responsive processes. For example, in ABA-containing media, *spl3* mutant produced longer primary roots and more adventitious roots than the WT (Fig. 5A–C), and showed hypersensitivity to drought (Fig. 5D, E) and osmotic stresses (Supplementary Fig. S11). Furthermore, stomatal closure in the *spl3* leaves was insensitive to ABA treatment (Fig. 5F, G). All these results demonstrate that SPL3 is involved in ABA signal transduction.

It was also found that many ABA signalling-associated genes were significantly down-regulated in the *spl3* leaves (Fig. 6). Among these genes, the physiological functions of several genes have been studied using rice overexpressing and/or antisense transgenic lines. *OsABI5* knockdown lines showed low spikelet fertility because of aberrant pollen development (Zou *et al.*, 2008). Similarly, the *spl3* mutant showed low spikelet fertility (Supplementary Fig. S2) and *OsABI5* was strongly down-regulated in the *spl3* leaves in response to ABA treatment (Fig. 6E), suggesting that *OsABI5* may be one of the important genes downstream of SPL3 in seed maturation. *OsbZIP23* (Fig. 6G) is considered a functional homologue of *Arabidopsis AREB* genes, based on physiological and phylogenetic studies (Xiang *et al.*, 2008). The transgenic lines overexpressing *OsbZIP23* showed tolerance to drought and high salinity stresses, and the T-DNA-insertion knockout lines showed sensitive phenotypes (Xiang *et al.*, 2008). *OsSAPK9* (Fig. 6I), a functional homologue of *Arabidopsis SnRK2* (Kobayashi *et al.*, 2005), was also significantly down-regulated in the *spl3* leaves. The *Arabidopsis* SnRK2 proteins participate in ABA signal transduction by directly phosphorylating ABA-responsive element (ABRE)-binding factors, including AREB1 (Fujita *et al.*, 2009). Similarly, OsSAPK9 phosphorylates one of the rice AREB homologues, TRAB1 (Kobayashi *et al.*, 2005). Thus, it appears that the hypersensitivity of the *spl3* mutant to drought and osmotic stresses may result from the down-regulation of *OsbZIP23* and *OsSAPK9*. It was also found that *OsRePRP2.1*, a positive regulator of ABA-dependent root growth inhibition (Tseng *et al.*, 2013), was substantially reduced in the *spl3* mutant (Fig. 6K), which probably contributes to the insensitivity of *spl3* roots to ABA-mediated root growth inhibition (Fig. 5A–C). However, the *spl3* seeds did not show higher germination rates in the presence of ABA, compared with the WT (Supplementary Fig. S12), indicating that SPL3 is involved in some, but not all of the ABA-responsive pathways, acting by indirectly regulating several ABA signalling-associated genes (Supplementary Fig. S15). Notably, *SPL3* expression was down-regulated in response to ABA treatment (Supplementary Fig. S7), suggesting that SPL3 contributes only to activating the early phase of ABA signalling when *SPL3* transcripts are abundant.

### SPL3 regulates ROS production via the ABA signalling pathway

In addition, it was found that this strong ABA insensitivity of the *spl3* mutant leads to the differential expression of *OsCAT* genes; expression of *OsCatA* and *OsCatC* is down-regulated in the *spl3* mutant. Catalases in plants can be classified into three classes by organ/tissue specificity and expression pattern (Willekens *et al.*, 1995; Mhamdi *et al.*, 2010). Class I CATs are highly expressed in photosynthetic tissues while class II catalases in vascular tissues, and class III catalases in seeds and reproductive tissues (Mhamdi *et al.*, 2010). Among the three OsCATs, OsCatC and OsCatA are classified class I and class II, respectively (Mhamdi *et al.* 2010). In *Arabidopsis*, the class I catalase *cat2* mutant showed a severe necrotic lesion phenotype under long day conditions (Queval *et al.*, 2007). Although the class II catalase *cat1* mutant did not show a necrotic phenotype, a *cat1 cat2* double mutant showed a much more severe lesion phenotype than the *cat1* single mutant (Mhamdi *et al.*, 2010), indicating that both CAT1 and CAT2 contribute to scavenging H_2_O_2_ in the leaves. Thus, it is possible that down-regulation of *OsCatA* and *OsCatC* in the *spl3* mutant causes increased concentration of ROS in the mature leaves (Fig. 2), which leads to cell-death lesion formation around the heading stage (Fig. 1). Similar to the *Arabidopsis cat2* mutant, the expressivity of the *spl3* mutation is somewhat photoperiodic-dependent; under CL conditions, the lesion mimic phenotype of the *spl3* mutant is very severe compared with the mutant grown under SDs (Fig. 2). Taking these results together, it was concluded that the mRNA levels of two *OsCAT* genes (*OsCatA* and *OsCatC*) and catalase activity in the *spl3* leaves were greatly suppressed, which probably leads to accumulation of excessive H_2_O_2_ and formation of lesions on the *spl3* leaves (Supplementary Fig. S15).

Similar to the three *CAT* genes in *Arabidopsis*, the three *OsCAT* genes were also induced by ABA treatment (Fig. 7). Thus, it seems that down-regulation of *OsCatA* and *OsCatC* in the *spl3* mutant is caused by an impairment of ABA signalling. In *Arabidopsis*, the MAPKK1-MAPK6 signalling cascade regulates metabolism of H_2_O_2_ scavenging by promoting *CAT1* expression (Xing *et al.*, 2008), similar to SPL3 function. A mutant of *MEKK1*, one of the *Arabidopsis* MAPKKKs, accumulates high levels of ROS and develops a local lesion mimic phenotype (Teige *et al.*, 2004). Furthermore, Ning *et al.* (2010) reported that a mutant of *DROUGHT-HYPERSENSITIVE MUTANT1* (*DSM1*), one of the OsMAPKKKs, accumulates excessive amounts of H_2_O_2_ under methyl viologen (MV)-induced oxidative stress, which is closely associated with down-regulation of two peroxidase genes, *POX22.3* and *POX8.1*. These results indicate that several MAPK cascades play critical roles in controlling H_2_O_2_ scavenging in plants. To the authors’ knowledge, either OsMAPKs or OsMAPKKs that act downstream of SPL3/OsMAPKKK1 and regulate *OsCAT* expression have not yet been identified, although several *MAPK* genes, such as *OsMAPK5*, *OsMAPK12*, *OsMAPK1*, and *OsMAPKK2*, are induced by ABA treatment (Xiong and Yang, 2003; You *et al.*, 2007).

### SPL3 promotes ABA and ethylene signalling pathways in leaf senescence

It was also found that the *spl3* mutant showed delayed leaf yellowing during both natural and dark-induced senescence (Fig. 5). Concurrently, *SPL3* expression increased during senescence (Supplementary Fig. S10), indicating that SPL3 contributes to promoting leaf senescence. In *Arabidopsis*, a few MAPK components have been revealed to be involved in the leaf senescence. Knockout mutants of *MAPKK9* and *MAPK6*, which are known to form a MAPK cascade together, showed commonly delayed senescence, indicating that the MAPKK9-MAPK6 cascade definitely promotes leaf senescence (Zhou *et al.*, 2009). MAPK6 accelerates SA-mediated leaf senescence by promoting the activity of NONEXPRESSOR OF PATHOGENESIS-RELATED GENE 1 (NPR1), which acts as a major component of SA-responsive signalling and as an inducer of leaf senescence (Ogawa *et al.*, 2005; Chai *et al.*, 2014). *Arabidopsis EDR1* also functions in leaf senescence; the leaves of the *edr1* mutant senesce early during ethylene-induced leaf senescence (Frye *et al.*, 2001; Tang and Innes, 2002). Recently, Matsuoka *et al.* (2015) reported that *Arabidopsis* MAPKKK18 is also involved in leaf senescence, because the loss-of-function mutant of MAPKKK18 showed a delayed senescence phenotype (Matsuoka *et al.*, 2015), indicating that MAPKKK18 positively regulates leaf senescence, similar to SPL3.

In the present study, it has been revealed how SPL3 exerts its function in the promotion of leaf senescence. When the rice plants enter the senescence phase, several ethylene- and ABA-associated genes are down-regulated in the *spl3* mutant (Supplementary Figs. S13 and S14), which probably leads to delaying leaf yellowing during natural and dark-induced senescence (Fig. 4). ABA and ethylene promote leaf yellowing (Gepstein and Thimann, 1981; Nooden, 1988), and *Arabidopsis* mutants or transgenic plants overexpressing genes that are related to ABA or ethylene signalling exhibited different senescence phenotypes (Kusaba *et al.*, 2013). In this study, it was found that *OsABI5*, *OsEIN2*, and *OsEIN3* were down-regulated in the *spl3* mutant during senescence (Supplementary Figs S13 and S14). Although previous work reported that ethylene synthesis is impaired in the *osedr1* mutant (Shen *et al.*, 2011), the finding that *OsEIN2* and *OsEIN3* are down-regulated in the *spl3* mutant indicates that both ethylene-synthesis and -signalling pathways are impaired in the *spl3* mutant during senescence. *Arabidopsis* homologues of these three genes were previously identified as senescence-promoting transcription factors. *Arabidopsis* ABI5 and EIN3 directly promote the expression of *ORESARA1* (*ORE1*; Kim *et al.*, 2014; Sakuraba *et al.*, 2014), which encodes a key senescence-promoting NAC transcription factor (Kim *et al.*, 2009*b*). EIN2 also activates *ORE1* expression by repressing the expression of *miR164*, which cleaves the *ORE1* mRNA; EIN2 also functions by a *miR164*-independent pathway (Kim *et al.*, 2009*b*; Li *et al.*, 2013). Furthermore, EIN3 also directly promotes the expression of *NAP* (Kim *et al.*, 2014), another key senescence-promoting NAC transcription factor (Guo and Gan, 2006). The rice homologue *OsNAP* was found to be down-regulated in the *spl3* mutant during senescence (Fig. 4E). *OsNAP* is induced by ABA treatment and directly activates ABA-responsive genes (Chen *et al.*, 2014; Liang *et al.*, 2014), similar to *AtNAP* function (Zhang and Gan, 2012; Yang *et al.*, 2014). Thus, it is probable that down-regulation of *OsNAP* expression in the *spl3* mutant was caused by an impairment of both ABA- and ethylene-responsive signalling pathways.

Here, it has been shown that SPL3 positively regulates the ABA-responsive signalling pathway, which affects several important processes, including root elongation, abiotic stress responses, stomatal closure, and leaf senescence (Figs 4 and 5; Supplementary Fig. S15). SPL3 indirectly promotes ABA and ethylene signalling (Fig. 6; Supplementary Figs S13 and 14), while suppressing both SA- and JA-associated defence signalling (Kim *et al.* 2009*a*; Shen *et al.*, 2011). Thus, SPL3 has a vital role in the crosstalk among important phytohormone signalling-associated processes, such as abiotic stress signalling, leaf senescence, and defence against pathogens. Because SPL3 is one of the OsMAPKKKs (Kim *et al.*, 2003), SPL3 likely regulates specific MAPKKs and MAPKs in the ethylene and ABA signalling pathways. Large-scale interactome analysis between OsMAPKKKs and OsMAPKKs will be necessary to reveal the SPL3-dependent MAPK cascade, as described in the previous study of OsMAPKKs and OsMAPKs (Singh *et al.*, 2012).

## Supplementary data

Supplementary data are available at *JXB* online.

Fig. S1. Lesion mimic phenotype of the *spl3* mutant is predominant in the tip region of leaf blades.

Fig. S2. Difference in plant height of the WT and *spl3* mutant.

Fig. S3. Agronomic traits of the *spl3* mutant.

Fig. S4. Amino acid sequence alignment of SPL3 and its homologues in other plant species.

Fig. S5. Complementation of the *spl3* mutant by transformation with *35S:SPL3*.

Fig. S6. Expression of *SPL3* in different organs of rice plants.

Fig. S7. Expression of *SPL3* under different abiotic stress conditions.

Fig. S8. Senescence phenotype of the *spl3* leaves under ABA, ACC, MeJA, and SA treatments.

Fig. S9. No difference of heading date in the WT and *spl3* mutant in the paddy field.

Fig. S10. Expression of *SPL3* during natural and dark-induced senescence.

Fig. S11. The *spl3* mutant is hypersensitive to osmotic stress.

Fig. S12. The effect of ABA on the germination rate of WT and *spl3* seeds.

Fig. S13. Altered expression of ABA-responsive genes in the *spl3* mutant during natural senescence.

Fig. S14. Altered expression of ET signalling- and synthesis-related genes in the *spl3* mutant during natural senescence.

Figure S15. Tentative model of the role of SPL3 in ABA-responsive signalling pathways.

Table S1. Primers used in this study.

## Accession numbers

Sequence data from this article can be found in the National Center for Biotechnology Information (NCBI) or GenBank/EMBL databases under the following accession numbers: *OsABI1*, Os09g0532400; *OsABI3*, Os01g091170; *OsABI4*, Os05g0351200; *OsABI5*, Os09g0456200; OsACS1, Os01g0978100; *OsACS2*, Os04g0578000; *OsAREB1*, Os06g0211200; *OsbZIP23*, Os02g0766700; *OsCatA*, Os02g0115700; *OsCatB*, Os06g0727200; *OsCatC*, Os03g0131200; *OsDSG1*, Os09g0434200; *OsDSR1*, Os10g0177200; *OsEIN2*, Os07g0155600; *OsEIN3*, Os03g0324200; *OsNAP*, Os03g0327800; *NYC1*, Os01g0227100; *OsRePRP2.1*, Os07g0418700; *OsSAPK6*, Os02g0551100; *OsSAPK8*, Os03g0764800; *OsSAPK9*, Os12g0586100; *SGR*, Os09g0532000; *OsUBQ5*, Os01g0328400; *SPL3*, Os03g0160100.

## Conflict of interest disclosure

The authors declare that they have no conflict of interest.

## Supplementary Material

Supplementary Data

## References

[CIT0001] Agrawal GK Iwahashi H Rakwal R 2003 Rice MAPKs. Biochemical and Biophysical Research Communications 302, 171–180. 12604328 10.1016/s0006-291x(03)00174-8

[CIT0002] Balague C Lin B Alcon C Flottes G Malmstrom S Kohler C Neuhaus G Pelletier G Gaymard F Roby D 2003 HLM1, an essential signalling component in the hypersensitive response, is a member of the cyclic nucleotide-gated channel ion channel family. Plant Cell 15, 365–379. 12566578 10.1105/tpc.006999 PMC141207

[CIT0003] Brossa R López-Carbonell M Jubany-Marí T Alegre L 2011 Interplay between abscisic acid and jasmonic acid and its role in water-oxidative stress in wild-type, ABA-deficient, JA-deficient, and ascorbate-deficient Arabidopsis plants. Journal of Plant Growth Regulation 30, 322–333.

[CIT0004] Chai J Liu J Zhou J Xing D 2014 Mitogen-activated protein kinase 6 regulates NPR1 gene expression and activation during leaf senescence induced by salicylic acid. Journal of Experimental Botany 65, 6513–6528. 25210078 10.1093/jxb/eru369

[CIT0005] Chen X Hao L Pan J Zheng X Jiang G Jin Y Gu Z Qian Q Zhai W Ma B 2012 SPL5, a cell death and defense-related gene, encodes a putative splicing factor 3b subunit 3 (SF3b3) in rice. Molecular Breeding 30, 939–949.

[CIT0006] Chen X Wang Y Lv B Li J Luo L Lu S Zhang X Ma H Ming F 2014 The NAC family transcription factor OsNAP confers abiotic stress response through the ABA pathway. Plant and Cell Physiology 55, 604–619. 24399239 10.1093/pcp/pct204

[CIT0007] Clarke SM Cristescu SM Miersch O Harren FJ Wasternack C Mur LA 2009 Jasmonates act with salicylic acid to confer basal thermotolerance in Arabidopsis thaliana. New Phytologist 182, 175–187. 19140948 10.1111/j.1469-8137.2008.02735.x

[CIT0008] Colcombet J Hirt H 2008 Arabidopsis MAPKs: a complex signalling network involved in multiple biological processes. Biochemical Journal 413, 217–226. 18570633 10.1042/BJ20080625

[CIT0009] Curtis MD Grossniklaus U 2003 A gateway cloning vector set for high-throughput functional analysis of genes in planta. Plant Physiology 133, 462–469. 14555774 10.1104/pp.103.027979 PMC523872

[CIT0010] De Smet I Zhang H Inze D Beeckman T 2006 A novel role for abscisic acid emerges from underground. Trends in Plant Science 11, 434–439. 16890475 10.1016/j.tplants.2006.07.003

[CIT0011] Dietrich RA Richberg MH Schmidt R Dean C Dangl JL 1997 A novel zinc finger protein is encoded by the Arabidopsis LSD1 gene and functions as a negative regulator of plant cell death. Cell 88, 685–694. 9054508 10.1016/s0092-8674(00)81911-x

[CIT0012] Frye CA Tang D Innes RW 2001 Negative regulation of defense responses in plants by a conserved MAPKK kinase. Proceedings of the National Academy of Sciences USA 98, 373–378. 10.1073/pnas.98.1.373 PMC14597 11114160

[CIT0013] Fujita Y Fujita M Satoh R 2005 AREB1 is a transcription activator of novel ABRE-dependent ABA signalling that enhances drought stress tolerance in Arabidopsis. Plant Cell 17, 3470–3488. 16284313 10.1105/tpc.105.035659 PMC1315382

[CIT0014] Fujita Y Nakashima K Yoshida T 2009 Three SnRK2 protein kinases are the main positive regulators of abscisic acid signalling in response to water stress in Arabidopsis. Plant and Cell Physiology 50, 2123–2132. 19880399 10.1093/pcp/pcp147

[CIT0015] Gao L Xiang CB 2008 The genetic locus At1g73660 encodes a putative MAPKKK and negatively regulates salt tolerance in Arabidopsis. Plant Molecular Biology 67, 125–134. 18299802 10.1007/s11103-008-9306-8

[CIT0016] Gepstein S Thimann KV 1981 The role of ethylene in the senescence of oat leaves. Plant Physiology 68, 349–354. 16661915 10.1104/pp.68.2.349 PMC427489

[CIT0017] Guo Y Gan S 2006 AtNAP, a NAC family transcription factor, has an important role in leaf senescence. Plant Journal 46, 601–612. 16640597 10.1111/j.1365-313X.2006.02723.x

[CIT0018] Hamel LP Nicole MC Sritubtim S 2006 Ancient signals: comparative genomics of plant MAPK and MAPKK gene families. Trends in Plant Science 11, 192–198. 16537113 10.1016/j.tplants.2006.02.007

[CIT0019] Han SH Sakuraba Y Koh HJ Paek NC 2012 Leaf variegation in the rice zebra2 mutant is caused by photoperiodic accumulation of tetra-cis-lycopene and singlet oxygen. Molecules and Cells 33, 87–97. 22134723 10.1007/s10059-012-2218-0 PMC3887748

[CIT0020] Hoisington DA Neuffer MG Walbot V 1982 Disease lesion mimics in maize. I. Effect of genetic background, temperature, developmental age, and wounding on necrotic spot formation with Les1. Developmental Biology 93, 381–388. 7141103 10.1016/0012-1606(82)90125-7

[CIT0021] Huang Y Li CY Qi Y Park S Gibson SI 2014 SIS8, a putative mitogen-activated protein kinase kinase kinase, regulates sugar-resistant seedling development in Arabidopsis. Plant Journal 77, 577–588. 24320620 10.1111/tpj.12404

[CIT0022] Iwamoto M Higo H Higo K 2000 Differential diurnal expression of rice catalase genes: the 5′-flanking region of *CatA* is not sufficient for circadian control. Plant Science 151, 39–46.

[CIT0023] Jeon JS Lee S Jung KH (2000) T-DNA insertional mutagenesis for functional genomics in rice. Plant Journal 22, 561–570. 10886776 10.1046/j.1365-313x.2000.00767.x

[CIT0024] Kieber JJ Rothenberg M Roman G Feldmann KA Ecker JR 1993 CTR1, a negative regulator of the ethylene response pathway in Arabidopsis, encodes a member of the raf family of protein kinases. Cell 72, 427–441. 8431946 10.1016/0092-8674(93)90119-b

[CIT0025] Kim JA Agrawal GK Rakwal R Han KS Kim KN Yun CH Heu S Park SY Lee YH Jwa NS 2003 Molecular cloning and mRNA expression analysis of a novel rice (Oryza sativa L.) MAPK kinase kinase, OsEDR1, an ortholog of Arabidopsis AtEDR1, reveal its role in defense/stress signalling pathways and development. Biochemical and Biophysical Research Communications 300, 868–876. 12559953 10.1016/s0006-291x(02)02944-3

[CIT0026] Kim JA Cho K Singh R (2009 *a* ) Rice OsACDR1 (Oryza sativa Accelerated Cell Death and Resistance 1) is a potential positive regulator of fungal disease resistance. Molecules and Cells 28, 431–439. 19904499 10.1007/s10059-009-0161-5

[CIT0027] Kim JH Woo HR Kim J Lim PO Lee IC Choi SH Hwang D Nam HG 2009 *b* . Trifurcate feed-forward regulation of age-dependent cell death involving miR164 in Arabidopsis. Science 323, 1053–1057. 19229035 10.1126/science.1166386

[CIT0028] Kim TW Michniewicz M Bergmann DC Wang ZY 2012 Brassinosteroid regulates stomatal development by GSK3-mediated inhibition of a MAPK pathway. Nature 482, 419–422. 22307275 10.1038/nature10794 PMC3292258

[CIT0029] Kim HJ Hong SH Kim YW 2014 Gene regulatory cascade of senescence-associated NAC transcription factors activated by ETHYLENE-INSENSITIVE2-mediated leaf senescence signalling in Arabidopsis. Journal of Experimental Botany 65, 4023–4036. 24659488 10.1093/jxb/eru112 PMC4106440

[CIT0030] Kobayashi Y Murata M Minami H Yamamoto S Kagaya Y Hobo T Yamamoto A Hattori T 2005 Abscisic acid-activated SNRK2 protein kinases function in the gene-regulation pathway of ABA signal transduction by phosphorylating ABA response element-binding factors. Plant Journal 44, 939–949. 16359387 10.1111/j.1365-313X.2005.02583.x

[CIT0031] Kusaba M Ito H Morita R 2007 Rice NON-YELLOW COLORING1 is involved in light-harvesting complex II and grana degradation during leaf senescence. Plant Cell , 19, 1362–1375. 17416733 10.1105/tpc.106.042911 PMC1913755

[CIT0032] Kusaba M Tanaka A Tanaka R 2013 Stay-green plants: what do they tell us about the molecular mechanism of leaf senescence. Photosynthesis Research 117, 221–234. 23771643 10.1007/s11120-013-9862-x

[CIT0033] Kusumi K Komori H Satoh H Iba K 2000 Characterization of a zebra mutant of rice with increased susceptibility to light stress. Plant and Cell Physiology 41, 158–164. 10795309 10.1093/pcp/41.2.158

[CIT0034] Lee SH Sakuraba Y Lee T Kim KY An G Lee HY Paek NC 2015 Mutation of Oryza sativa CORONATINE INSENSITIVE 1b (OsCOI1b) delays leaf senescence. Journal of Integrative Plant Biology 57, 562–576. 25146897 10.1111/jipb.12276

[CIT0035] Lei G Shen M Li ZG 2011 EIN2 regulates salt stress response and interacts with a MA3 domain-containing protein ECIP1 in Arabidopsis. Plant, Cell and Environment 34, 1678–1692. 10.1111/j.1365-3040.2011.02363.x 21631530

[CIT0036] Li J Pandeya D Nath K 2010 ZEBRA-NECROSIS, a thylakoid-bound protein, is critical for the photoprotection of developing chloroplasts during early leaf development. Plant Journal 62, 713–725. 20202171 10.1111/j.1365-313X.2010.04183.x

[CIT0037] Li Z Peng J Wen X Guo H 2013 Ethylene-insensitive3 is a senescence-associated gene that accelerates age-dependent leaf senescence by directly repressing miR164 transcription in Arabidopsis. Plant Cell 25, 3311–3328. 24064769 10.1105/tpc.113.113340 PMC3809534

[CIT0038] Liang C Wang Y Zhu Y 2014 OsNAP connects abscisic acid and leaf senescence by fine-tuning abscisic acid biosynthesis and directly targeting senescence-associated genes in rice. Proceedings of the National Academy of Sciences USA 111, 10013–10018. 10.1073/pnas.1321568111 PMC4103337 24951508

[CIT0039] Ligterink W 2000 MAP kinases in plant signal transduction: how many, and what for? Results and Problems in Cell Differentiation 27, 11–27. 10533195 10.1007/978-3-540-49166-8_2

[CIT0040] Lim JH Yang HJ Jung KH Yoo SC Paek NC 2014 Quantitative trait locus mapping and candidate gene analysis for plant architecture traits using whole genome re-sequencing in rice. Molecules and Cells 37, 149–160. 24599000 10.14348/molcells.2014.2336 PMC3935628

[CIT0041] Livak KJ Schmittgen TD 2001 Analysis of relative gene expression data using real-time quantitative PCR and the 2^-∆∆C^T method. Methods 25, 402–408. 11846609 10.1006/meth.2001.1262

[CIT0042] Lorrain S Lin B Auriac MC Kroj T Saindrenan P Nicole M Balagué C Roby D 2004 VASCULAR ASSOCIATED DEATH1, a novel GRAM domain-containing protein, is a regulator of cell death and defense responses in vascular tissues. Plant Cell 16, 2217–2232. 15269331 10.1105/tpc.104.022038 PMC519209

[CIT0043] Matsuoka D Yasufuku T Furuya T Nanmori T (2015) An abscisic acid inducible Arabidopsis MAPKKK, MAPKKK18 regulates leaf senescence via its kinase activity. Plant Molecular Biology 87, 565–575. 25680457 10.1007/s11103-015-0295-0

[CIT0044] Mhamdi A Queval G Chaouch S Vanderauwera S Van Breusegem F Noctor G 2010 Catalase function in plants: a focus on Arabidopsis mutants as stress-mimic models. Journal of Experimental Botany 61, 4197–4220. 20876333 10.1093/jxb/erq282

[CIT0045] Mosher S Moeder W Nishimura N Jikumaru Y Joo SH Urquhart W Klessig DF Kim SK Nambara E Yoshioka K 2010 The lesion-mimic mutant cpr22 shows alterations in abscisic acid signalling and abscisic acid insensitivity in a salicylic acid-dependent manner. Plant Physiology 152, 1901–1913. 20164209 10.1104/pp.109.152603 PMC2850030

[CIT0046] Nakashima K Yamaguchi-Shinozaki K 2013 ABA signalling in stress-response and seed development. Plant Cell Reports 32, 959–970. 23535869 10.1007/s00299-013-1418-1

[CIT0047] Ning J Li X Hicks LM Xiong L 2010 A Raf-like MAPKKK gene DSM1 mediates drought resistance through reactive oxygen species scavenging in rice. Plant Physiology 152, 876–890. 20007444 10.1104/pp.109.149856 PMC2815886

[CIT0048] Nooden LD 1988 Postlude and prospects. Senescence and aging in plants (Academic Press), pp 499–517.

[CIT0049] Noutoshi Y Kuromori T Wada T 2006 Loss of Necrotic Spotted Lesions 1 associates with cell death and defense responses in Arabidopsis thaliana. Plant Molecular Biology 62, 29–42. 16900325 10.1007/s11103-006-9001-6

[CIT0050] Ogawa T Pan L Kawai-Yamada M 2005 Functional analysis of Arabidopsis ethylene-responsive element binding protein conferring resistance to Bax and abiotic stress-induced plant cell death. Plant Physiology 138, 1436–1445. 15980186 10.1104/pp.105.063586 PMC1176415

[CIT0051] Park GG Park JJ Yoon J Yu SN An G 2010 A RING finger E3 ligase gene, Oryza sativa Delayed Seed Germination 1 (OsDSG1), controls seed germination and stress responses in rice. Plant Molecular Biology 74, 467–478. 20878348 10.1007/s11103-010-9687-3

[CIT0052] Park SY Yu JW Park JS 2007 The senescence-induced staygreen protein regulates chlorophyll degradation. Plant Cell 19, 1649–1664. 17513504 10.1105/tpc.106.044891 PMC1913741

[CIT0053] Porra R Thompson W Kriedemann P 1989 Determination of accurate extinction coefficients and simultaneous equations for assaying chlorophylls a and b extracted with four different solvents: verification of the concentration of chlorophyll standards by atomic absorption spectroscopy. Biochimica et Biophysica Acta-Bioenergetics 975, 384–394.

[CIT0054] Qiao Y Jiang W Lee J 2010 SPL28 encodes a clathrin-associated adaptor protein complex 1, medium subunit micro 1 (AP1M1) and is responsible for spotted leaf and early senescence in rice (Oryza sativa). New Phytologist 185, 258–274. 19825016 10.1111/j.1469-8137.2009.03047.x

[CIT0055] Queval G Issakidis‐Bourguet E Hoeberichts FA Vandorpe M Gakière B Vanacker H Miginiac-Maslow M Van Breusegem F Noctor G 2007 Conditional oxidative stress responses in the Arabidopsis photorespiratory mutant cat2 demonstrate that redox state is a key modulator of daylength-dependent gene expression, and define photoperiod as a crucial factor in the regulation of H2O2‐induced cell death. Plant Journal 52, 640–657. 17877712 10.1111/j.1365-313X.2007.03263.x

[CIT0056] Radin JW 1984 Stomatal responses to water stress and to abscisic acid in phosphorus-deficient cotton plants. Plant Physiology 76, 392–394. 16663851 10.1104/pp.76.2.392 PMC1064297

[CIT0057] Rao KP Richa T Kumar K Raghuram B Sinha AK 2010 In silico analysis reveals 75 members of mitogen-activated protein kinase kinase kinase gene family in rice. DNA Research 17, 139–153. 20395279 10.1093/dnares/dsq011 PMC2885274

[CIT0058] Robert-Seilaniantz A Navarro L Bari R Jones JD 2007 Pathological hormone imbalances. Current Opinion in Plant Biology 10, 372–379. 17646123 10.1016/j.pbi.2007.06.003

[CIT0059] Rostoks N Schmierer D Mudie S Drader T Brueggeman R Caldwell DG Waugh R Kleinhofs A 2006 Barley necrotic locus nec1 encodes the cyclic nucleotide-gated ion channel 4 homologous to the Arabidopsis HLM1. Molecular Genetics and Genomics 275, 159–168. 16341885 10.1007/s00438-005-0073-9

[CIT0060] Sakuraba Y Rahman ML Cho SH Kim YS Koh HJ Yoo SC Paek NC 2013 The rice faded green leaf locus encodes protochlorophyllide oxidoreductase B and is essential for chlorophyll synthesis under high light conditions. Plant Journal 74, 122–133. 23289852 10.1111/tpj.12110

[CIT0061] Sakuraba Y Jeong J Kang MY Kim J Paek NC Choi G 2014 Phytochrome-interacting transcription factors PIF4 and PIF5 induce leaf senescence in Arabidopsis. Nature Communications 5, 4636. 10.1038/ncomms5636 25119965

[CIT0062] Schaeffer HJ Weber MJ 1999 Mitogen-activated protein kinases: specific messages from ubiquitous messengers. Molecular and Cell Biology 19, 2435–2444. 10.1128/mcb.19.4.2435 PMC84036 10082509

[CIT0063] Shen X Liu H Yuan B Li X Xu C Wang S 2011 OsEDR1 negatively regulates rice bacterial resistance via activation of ethylene biosynthesis. Plant, Cell and Environment 34, 179–191. 10.1111/j.1365-3040.2010.02219.x 20807375

[CIT0064] Shirsekar GS Vega-Sanchez ME Bordeos A 2014 Identification and characterization of suppressor mutants of spl11-mediated cell death in rice. Molecular Plant-Microbe Interactions 27, 528–536. 24794921 10.1094/MPMI-08-13-0259-R

[CIT0065] Singh R Lee MO Lee JE 2012 Rice mitogen-activated protein kinase interactome analysis using the yeast two-hybrid system. Plant Physiology 160, 477–487. 22786887 10.1104/pp.112.200071 PMC3440221

[CIT0066] Takahashi Y Soyano T Kosetsu K Sasabe M Machida Y 2010 HINKEL kinesin, ANP MAPKKKs and MKK6/ANQ MAPKK, which phosphorylates and activates MPK4 MAPK, constitute a pathway that is required for cytokinesis in Arabidopsis thaliana. Plant and Cell Physiology 51, 1766–1776. 20802223 10.1093/pcp/pcq135 PMC2951530

[CIT0067] Tang D Innes RW 2002 Overexpression of a kinase-deficient form of the EDR1 gene enhances powdery mildew resistance and ethylene-induced senescence in Arabidopsis. Plant Journal 32, 975–983. 12492839 10.1046/j.1365-313x.2002.01482.x

[CIT0068] Tang D Christiansen KM Innes RW 2005 Regulation of plant disease resistance, stress responses, cell death, and ethylene signalling in Arabidopsis by the EDR1 protein kinase. Plant Physiology 138, 1018–1026. 15894742 10.1104/pp.105.060400 PMC1150416

[CIT0069] Teige M Scheikl E Eulgem T Doczi R Ichimura K Shinozaki K Dangl JL Hirt H 2004 The MKK2 pathway mediates cold and salt stress signalling in Arabidopsis. Molecular Cell 15, 141–152. 15225555 10.1016/j.molcel.2004.06.023

[CIT0070] Tseng IC Hong CY Yu SM Ho TH 2013 Abscisic acid- and stress-induced highly proline-rich glycoproteins regulate root growth in rice. Plant Physiology 163, 118–134. 23886623 10.1104/pp.113.217547 PMC3762635

[CIT0071] Wang L Pei Z Tian Y He C 2005 OsLSD1, a rice zinc finger protein, regulates programmed cell death and callus differentiation. Molecular Plant-Microbe Interactions 18, 375–384. 15915636 10.1094/MPMI-18-0375

[CIT0072] Widmann C Gibson S Jarpe MB Johnson GL 1999 Mitogen-activated protein kinase: conservation of a three-kinase module from yeast to human. Physiological Reviews 79, 143–180. 9922370 10.1152/physrev.1999.79.1.143

[CIT0073] Willekens H Inzé D Van Montagu M Van Camp W 1995 Catalases in plants. Molecular Breeding 1, 207–228.

[CIT0074] Wolter M Hollricher K Salamini F Schulze-Lefert P 1993 The mlo resistance alleles to powdery mildew infection in barley trigger a developmentally controlled defence mimic phenotype. Molecular and General Genetics 239, 122–128. 8510641 10.1007/BF00281610

[CIT0075] Wu C Bordeos A Madamba MR Baraoidan M Ramos M Wang GL Leach JE Leung H 2008 Rice lesion mimic mutants with enhanced resistance to diseases. Molecular Genetics and Genomics 279, 605–619. 18357468 10.1007/s00438-008-0337-2

[CIT0076] Xiang Y Tang N Du H Ye H Xiong L 2008 Characterization of OsbZIP23 as a key player of the basic zipper transcription factor family for conferring abscisic acid sensitivity and salinity and drought tolerance in rice. Plant Physiology 148, 1938–1952. 18931143 10.1104/pp.108.128199 PMC2593664

[CIT0077] Xing Y Jia W Zhang J 2007 AtMEK1 mediates stress-induced gene expression of CAT1 catalase by triggering H2O2 production in Arabidopsis. Journal of Experimental Botany 58, 2969–2981. 17728292 10.1093/jxb/erm144

[CIT0078] Xing Y Jia W Zhang J 2008 AtMKK1 mediates ABA-induced CAT1 expression and H2O2 production via AtMPK6-coupled signalling in Arabidopsis. Plant Journal 54, 440–451. 18248592 10.1111/j.1365-313X.2008.03433.x

[CIT0079] Xiong L Yang Y 2003 Disease resistance and abiotic stress tolerance in rice are inversely modulated by an abscisic acid-inducible mitogen-activated protein kinase. Plant Cell 15, 745–759. 12615946 10.1105/tpc.008714 PMC150027

[CIT0080] Yamanouchi U Yano M Lin H Ashikari M Yamada K 2002 A rice spotted leaf gene, Spl7, encodes a heat stress transcription factor protein. Proceedings of the National Academy of Sciences USA 99, 7530–7535. 10.1073/pnas.112209199 PMC124274 12032317

[CIT0081] Yang J Worley E Udvardi M 2014 A NAP-AAO3 regulatory module promotes chlorophyll degradation via ABA biosynthesis in Arabidopsis leaves. Plant Cell 26, 4862–4874. 25516602 10.1105/tpc.114.133769 PMC4311216

[CIT0082] Yoo SD Sheen J 2008 MAPK signalling in plant hormone ethylene signal transduction. Plant Signalling and Behavior 3, 848–849. 10.4161/psb.3.10.5995 PMC2634393 19704518

[CIT0083] Yoshida T Fujita Y Sayama H Kidokoro S Maruyama K Mizoi J Shinozaki K Yamaguchi-Shinozaki K 2010 AREB1, AREB2, and ABF3 are master transcription factors that cooperatively regulate ABRE-dependent ABA signalling involved in drought stress tolerance and require ABA for full activation. Plant Journal 61, 672–685. 19947981 10.1111/j.1365-313X.2009.04092.x

[CIT0084] Yoshimura A Ideta O Iwata N 1997 Linkage map of phenotype and RFLP markers in rice. Plant Molecular Biology 35, 49–60. 9291959

[CIT0085] You MK Oh S Ok SH Cho SK Shin HY Jeung JU Shin JS 2007 Identification of putative MAPK kinases in Oryza minuta and O. sativa responsive to biotic stresses. Molecules and Cells 23, 108–114. 17464219

[CIT0086] Zeng LR Qu S Bordeos A Yang C Baraoidan M Yan H Xie Q Nahm BH Leung H Wang GL 2004 Spotted leaf11, a negative regulator of plant cell death and defense, encodes a U-box/armadillo repeat protein endowed with E3 ubiquitin ligase activity. Plant Cell 16, 2795–2808. 15377756 10.1105/tpc.104.025171 PMC520972

[CIT0087] Zhang K Gan SS 2012 An abscisic acid-AtNAP transcription factor-SAG113 protein phosphatase 2C regulatory chain for controlling dehydration in senescing Arabidopsis leaves. Plant Physiology 158, 961–969. 22184656 10.1104/pp.111.190876 PMC3271781

[CIT0088] Zhou C Cai Z Guo Y Gan S 2009 An Arabidopsis mitogen-activated protein kinase cascade, MKK9-MPK6, plays a role in leaf senescence. Plant Physiology 150, 167–177. 19251906 10.1104/pp.108.133439 PMC2675715

[CIT0089] Zhou J Zhang H Yang Y Zhang Z Zhang H Hu X Chen J Wang XC Huang R 2008 Abscisic acid regulates TSRF1-mediated resistance to Ralstonia solanacearum by modifying the expression of GCC box-containing genes in tobacco. Journal of Experimental Botany 59, 645–652. 18252700 10.1093/jxb/erm353

[CIT0090] Zou M Guan Y Ren H Zhang F Chen F 2008 A bZIP transcription factor, OsABI5, is involved in rice fertility and stress tolerance. Plant Molecular Biology 66, 675–683. 18236009 10.1007/s11103-008-9298-4

