# Supplementary material for: Mutation of SPOTTED LEAF3 (SPL3) impairs abscisic acid-responsive signalling and delays leaf senescence in rice
Source: J Exp Bot. 2015 Aug 14;66(22):7045–59. doi: 10.1093/jxb/erv401 (PMC4765782; doi:10.1093/jxb/erv401)
Supplement: Supplementary Data [file supp_66_22_7045__index.html]

Mutation of SPOTTED LEAF3 (SPL3) impairs abscisic acid-responsive signalling and delays leaf senescence in rice — Mutation of SPOTTED LEAF3 (SPL3) impairs abscisic acid-responsive signalling and delays leaf senescence in rice — Supplementary Data 

# Mutation of *SPOTTED LEAF3* (*SPL3*) impairs abscisic acid-responsive signalling and delays leaf senescence in rice

## Supplementary Data

Data files

- Supplementary Data - Supplementary Data
