# Supplementary material for: Mutation of SPOTTED LEAF3 (SPL3) impairs abscisic acid-responsive signalling and delays leaf senescence in rice
Source: J Exp Bot. 2015 Aug 14;66(22):7045–59. doi: 10.1093/jxb/erv401 (PMC4765782; doi:10.1093/jxb/erv401)
Supplement: Supplementary Data [file supp_erv401_jexbot154880_file001.pdf]

Supplementary Data at *JXB* online – Wang *et al.* (2015)

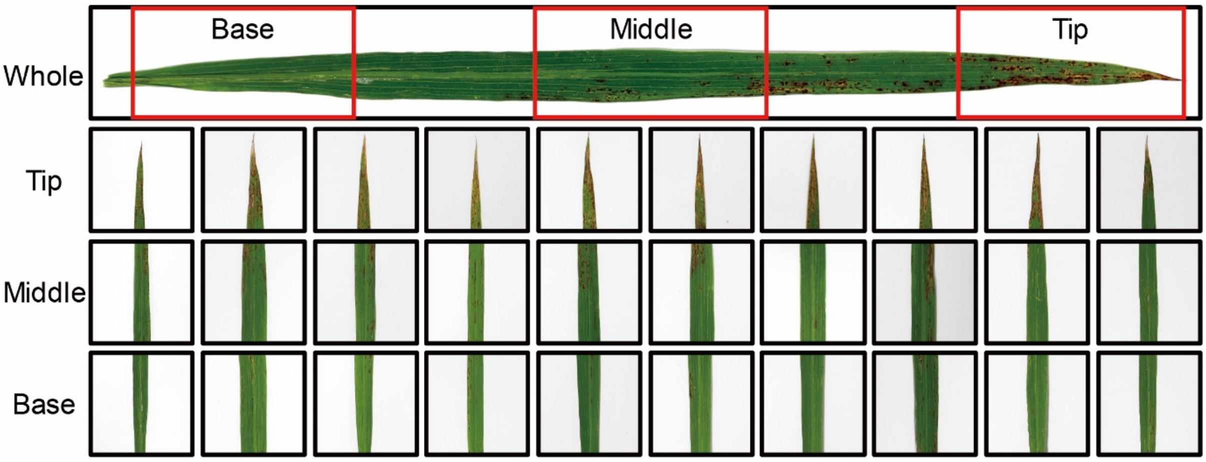

Supplementary Fig. S1. Lesion mimic phenotype of *spl3* leaves is predominant in the tip region of leaf blades.

The 3rd leaf blades (from top) of main culms from 10 independent *spl3* mutant plants were sampled at 90 DAS grown in the paddy field.

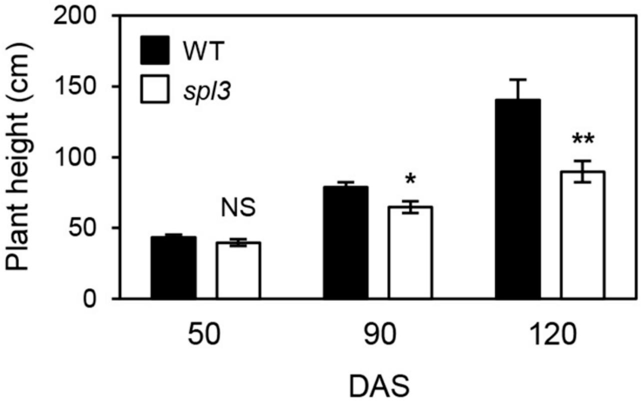

Supplementary Fig. S2. Difference in plant height of WT and *spl3* mutant.

WT (cv. Norin8) and *spl3* mutant were grown in the paddy field, and their heights at 50, 90, and 120 d after sowing (DAS) were compared. Mean and SD values were obtained from more than ten independent plants grown in the paddy field in 2014. Statistical analysis used Student's *t*-test (* $P < 0.05$, ** $P < 0.01$). NS, no significance.

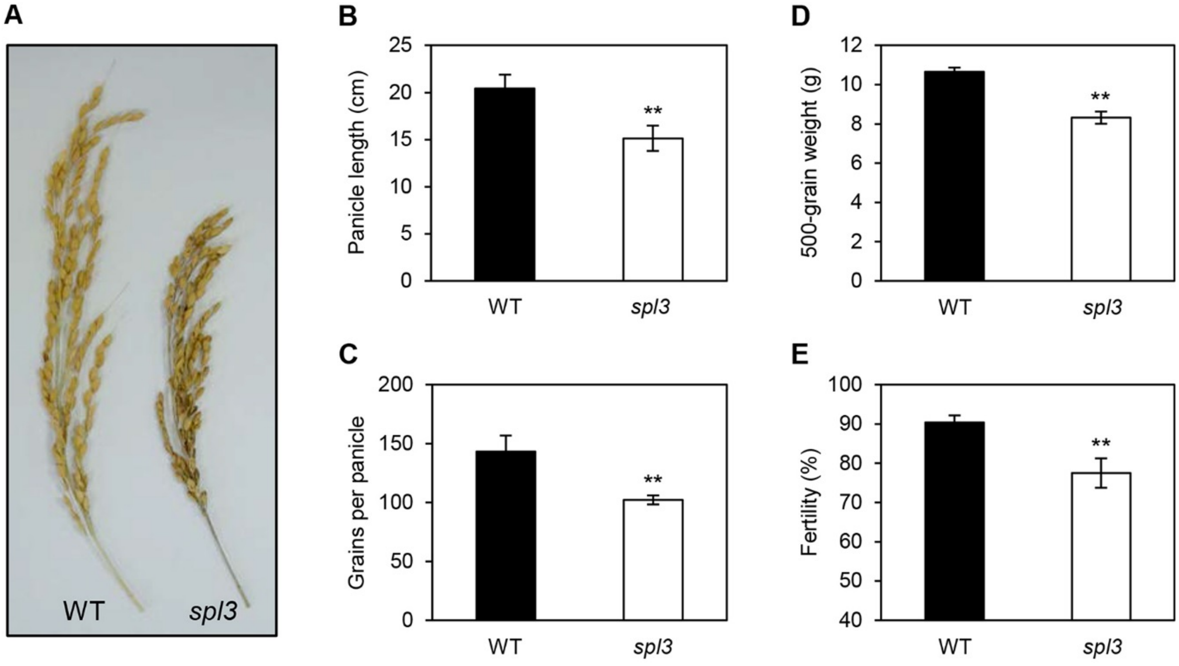

Supplementary Fig. S3. Agronomic traits of *spl3* mutant.

Panicles (A), panicle length (B), number of grains per panicle (C), 500-grain weight (D), and spikelet fertility (E) were compared between WT and *spl3* mutant. Mean and SD values were obtained from more than ten replicates from the plants grown in the paddy field in 2014. Statistical analysis used Student's *t*-test (** $P < 0.01$).

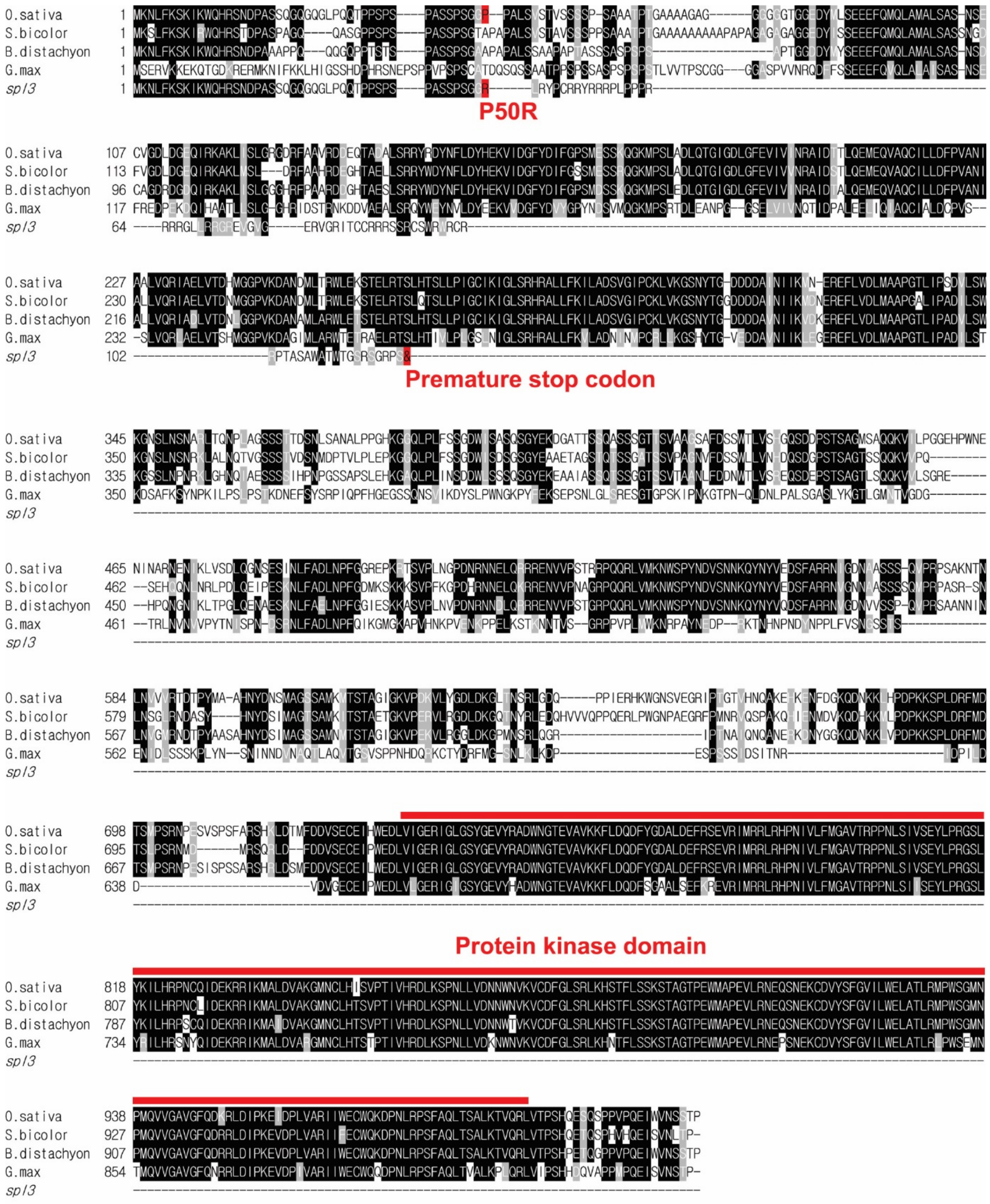

Supplementary Fig. S4. Amino acid sequence alignment of SPL3 and its homologs in other plant species.

The substitution point by 1-bp deletion in the *spl3* allele (P50R), a premature stop codon, and a protein kinase domain are indicated in red.

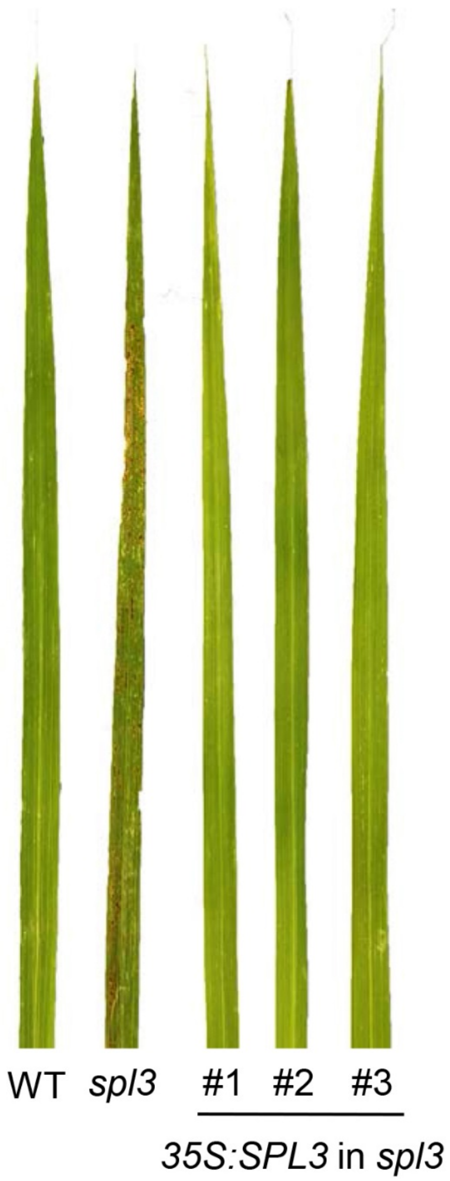

Supplementary Fig. S5. Complementation of *spl3* mutant by transformation with *35S:SPL3*.

Phenotypes of 2-month-old WT, *spl3* mutant, and three independent 3*5S:SPL3/spl3* transgenic plants grown in LD conditions are shown.

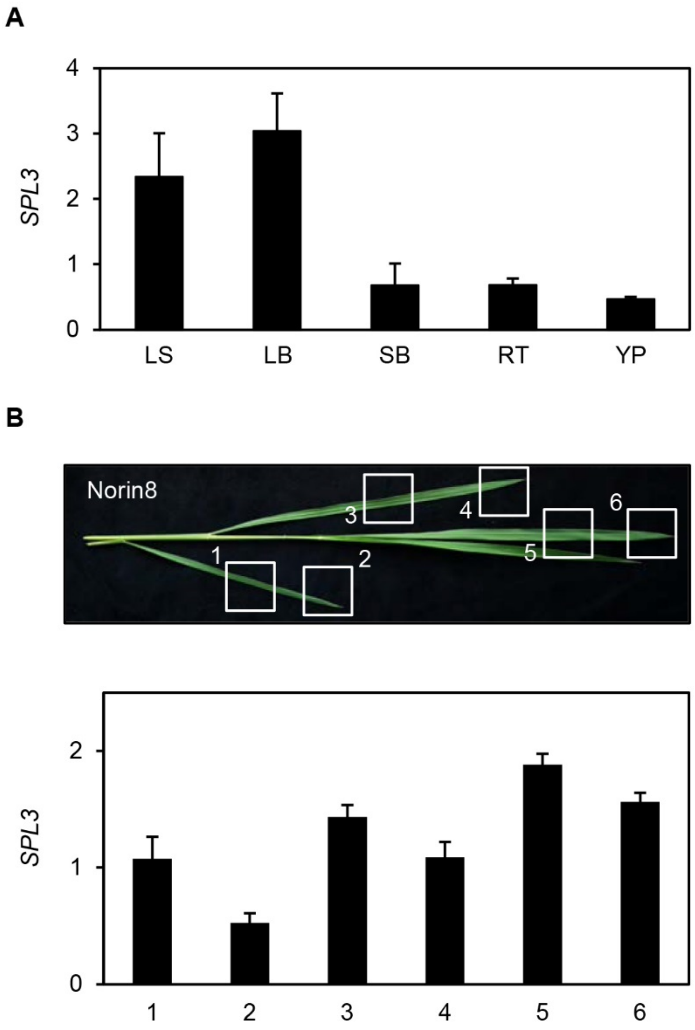

Supplementary Fig. S6. Expression of *SPL3* in different organs of rice plants.

(A) *SPL3* expression in various tissues. Total RNA was extracted from leaf sheath (LS), leaf blade (LB), shoot base (SB), or root (RT) tissues of 2-week-old seedlings, or young panicle (YP) tissues, which were collected before emergence from the main stem. (B) *SPL3* expression in different parts of one tiller. The middle (older-1, 3, 5-younger) and distal (older-2, 4, 6=younger) parts of the three leaves from the main stem of 2-month-old WT plants were used to determine the transcript levels of *SPL3*. RT-qPCR was used to measure the relative transcript levels of *SPL3*, which were normalized to the transcript levels of *OsUBQ5*. Mean and SD values were obtained from more than three biological replicates. These experiments were repeated two times with similar results. HT, hour(s) of treatment.

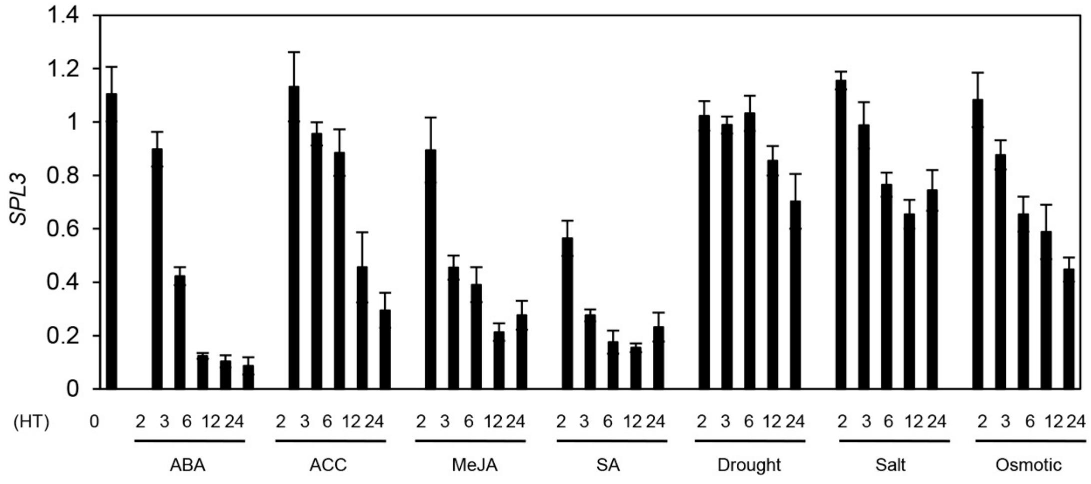

Supplementary Fig. S7. Expression of *SPL3* under different abiotic stress conditions. Two-week-old WT (cv. Norin8) seedlings were treated with ABA (100 μM), ACC (10 mM), MeJA (100 μM), SA (100 μM), drought, NaCl (150 mM), and mannitol (500 mM). Treated seedlings were sampled at 2, 3, 6, 12, and 24 h after each treatment. RT-qPCR was used to measure the relative transcript levels of *SPL3,* which were normalized to the transcript levels of *OsUBQ5*. Mean and SD values were obtained from more than three biological replicates. HT, hours of treatment.

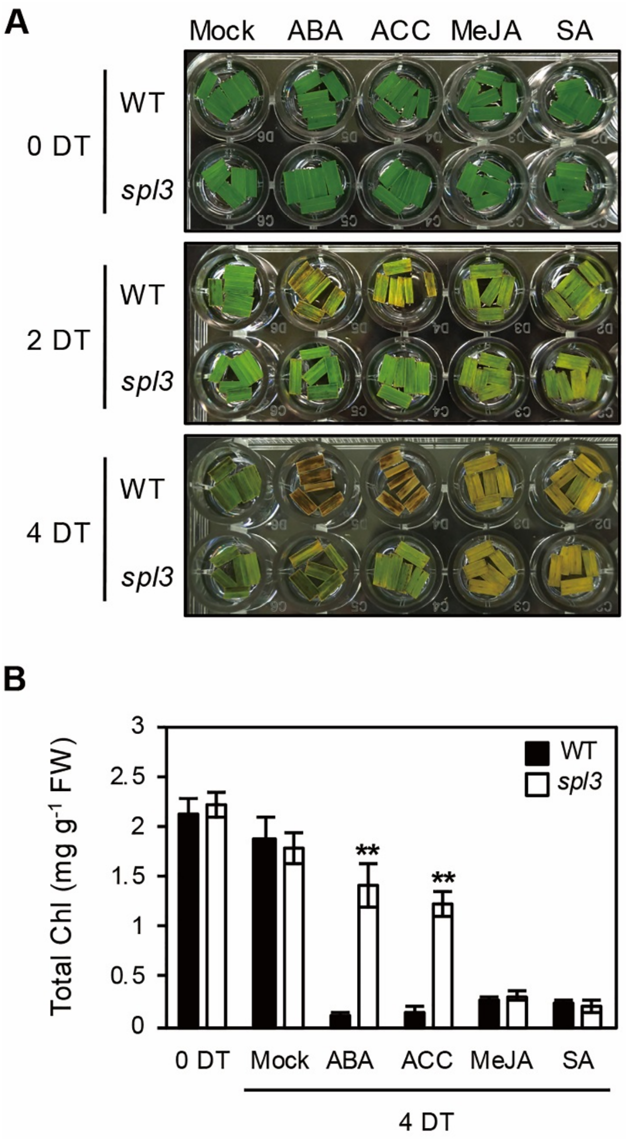

Supplementary Fig. S8. Senescence phenotype of *spl3* leaves under ABA, ACC, MeJA, and SA treatments.

The leaf discs from 1-month-old WT and *spl3* plants grown under LD conditions were floated on 3 mM MES buffer (pH 5.8) supplemented with 50 μM ABA, 10 mM ACC, 100 μM MeJA, or 100 μM SA, and incubated for 4 d under continuous light. Changes in leaf color (A) and total Chl level (B) are shown. (B) Black and white bars indicate WT and *spl3*, respectively. Statistical analysis used Student's *t*-test (** $P < 0.01$). DT, day(s) of treatment.

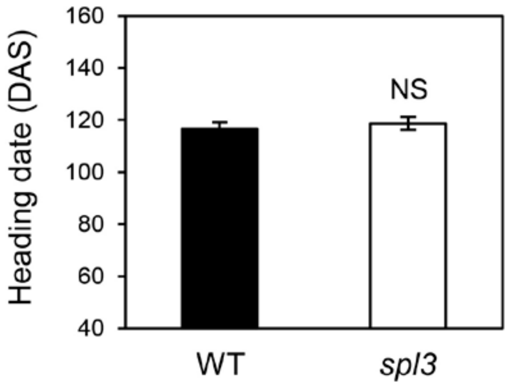

Supplementary Fig. S9. No difference of heading date in WT and *spl3* mutant in the paddy field. Mean and SD values were obtained from more than ten independent plants grown in the paddy field in 2014. Statistical analysis used Student's *t*-test. NS, no significance; DAS, days after sowing.

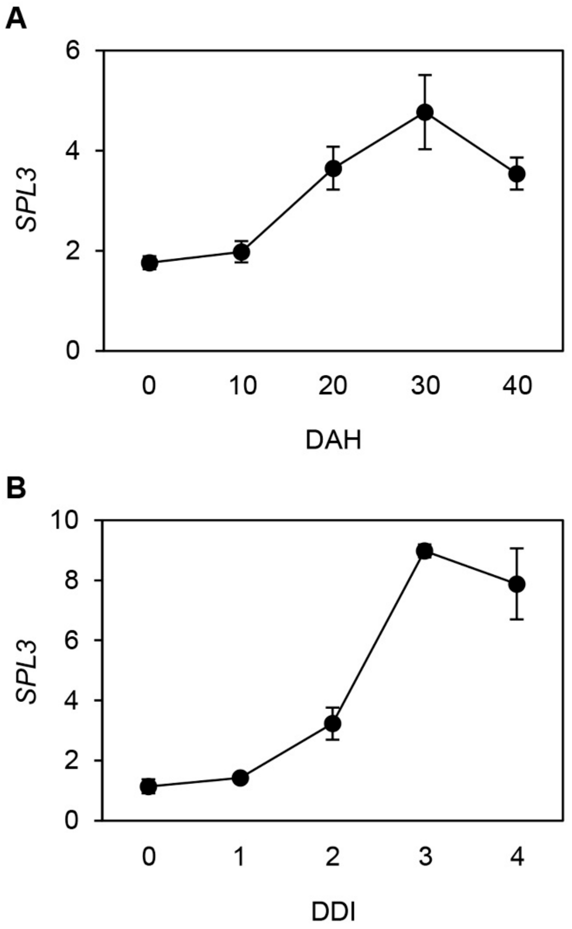

Supplementary Fig. S10. Expression of *SPL3* during natural and dark-induced senescence.
For the expression levels of *SPL3* during natural senescence (A), the 3rd leaves of the main culm were sampled. During dark-induced senescence (B), 1-month-old plants grown under LD were incubated in complete darkness, and the 2nd leaves were sampled. RT-qPCR was used to measure the relative transcript levels of *SPL3*, which were normalized to the transcript levels of *OsUBQ5*. Mean and SD values were obtained from more than three biological replicates. These experiments were repeated two times with similar results. DAH, days after heading. DDI, days of dark incubation.

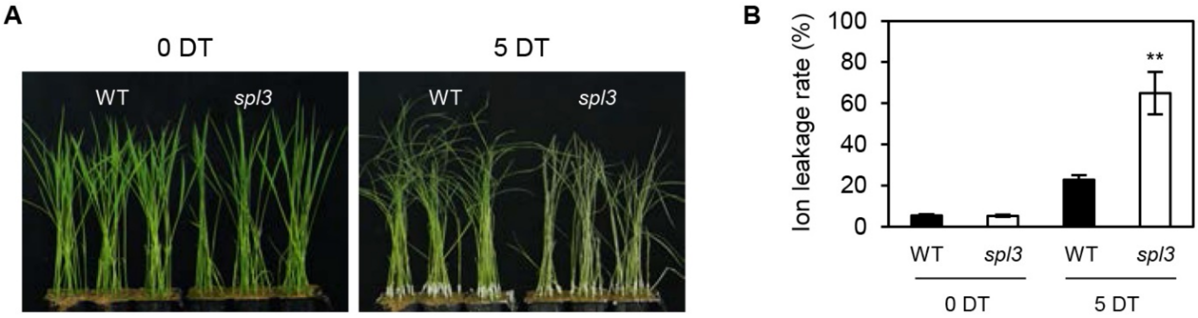

Supplementary Fig. S11. *spl3* mutant is hypersensitive to osmotic stress.

The 4-week-old WT and *spl3* plants grown under LD conditions were transferred to 500 mM mannitol and incubated for 5 d. Changes in phenotype (A) and ion leakages rates (B) at 0 d (control) and 5 d of treatment (5 DT) are shown. Statistical analysis used Student's *t*-test (** $P < 0.01$). DT, day(s) of treatment.

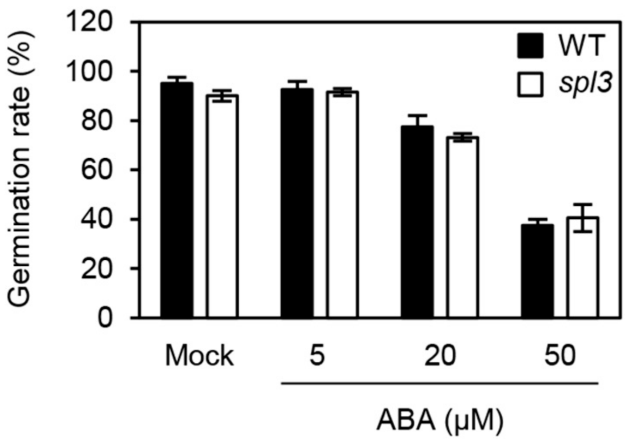

Supplementary Fig. S12. The effect of ABA on the germination rate of WT and *spl3* seeds.

Seeds of WT and *spl3*, harvested in the autumn of 2014, were incubated for 7 d in MES buffer (pH5.8) supplemented with 0 (mock control), 5, 20, and 50 μM ABA to check the germination rate.

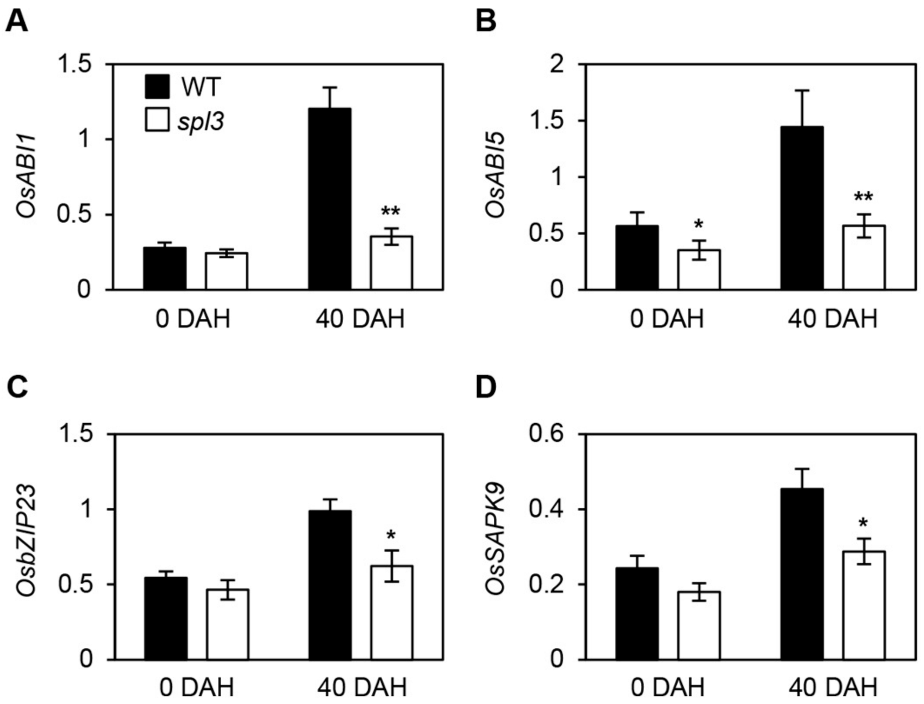

Supplementary Fig. S13. Altered expression of ABA-responsive genes in *spl3* mutant during natural senescence.

RT-qPCR was used to measure the relative transcript levels of *OsABI1* (A), *OsABI5* (B), *OsbZIP23* (C), and *OsSAPK9* (D) at 0 and 40 DAH, and transcript levels were normalized to the transcript levels of *OsUBQ5*. Mean and SD values were obtained from more than three biological replicates. These experiments were repeated two times with similar results. Statistical analysis used Student's *t*-test (* $P < 0.05$, ** $P < 0.01$). DAH, days after heading.

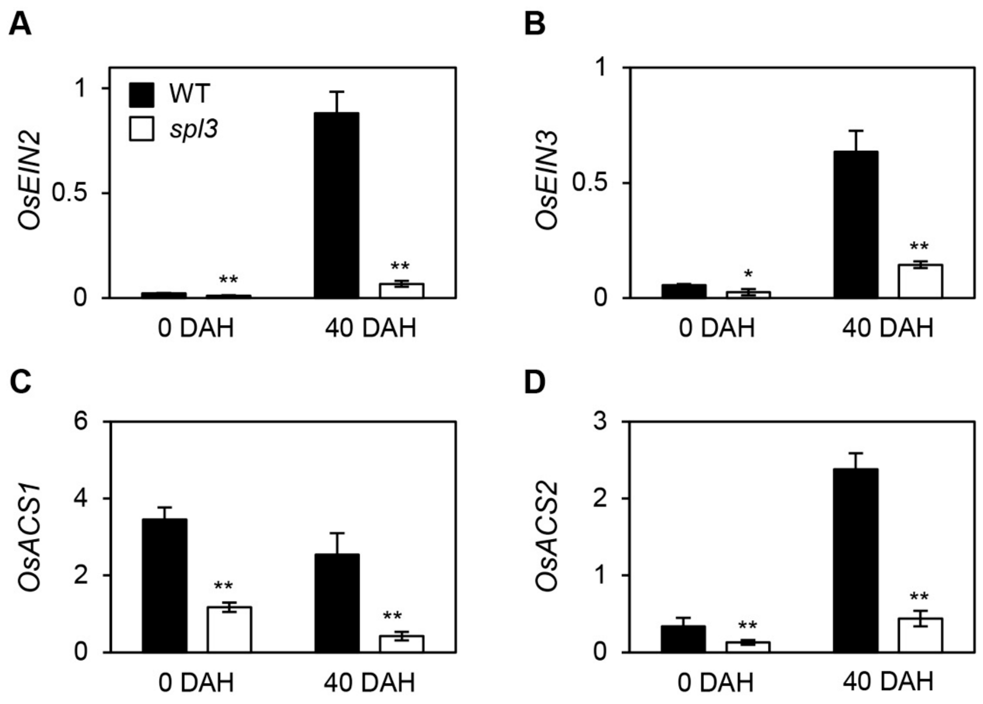

Supplementary Fig. S14. Altered expression of ET signaling- and synthesis-related genes in *spl3* mutant during natural senescence.

RT-qPCR was used to measure the relative transcript levels of *OsEIN2* (A), *OsEIN3* (B), *OsACS1* (C), and *OsACS2* (D) at 0 and 40 DAH, and transcript levels were normalized to the transcript levels of *OsUBQ5*. Mean and SD values were obtained from more than three biological replicates. These experiments were repeated two times with similar results. Statistical analysis used Student's *t*-test (* $P < 0.05$, ** $P < 0.01$). DAH, days after heading.

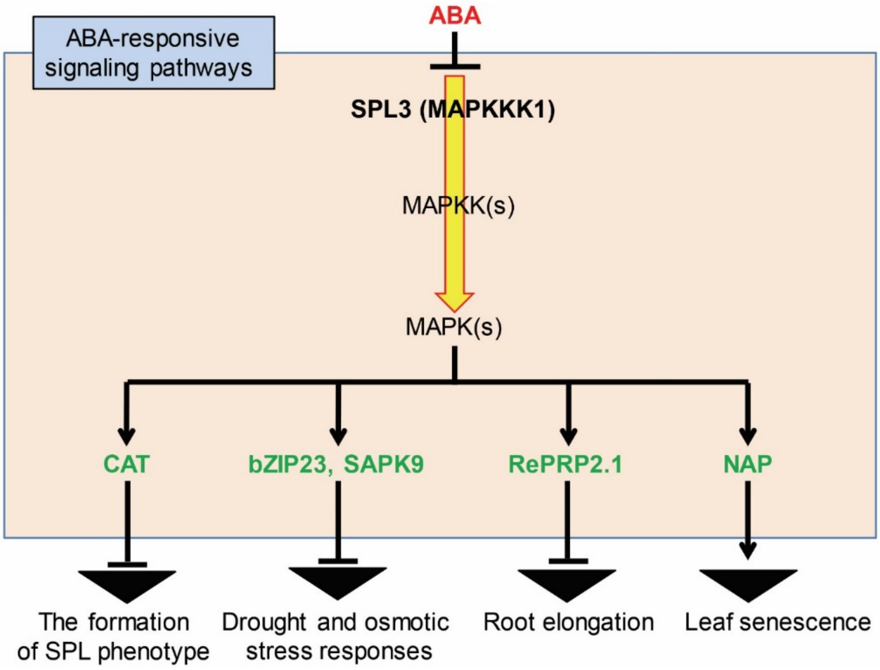

Supplementary Fig. S15. Tentative model of SPL3 role in ABA-responsive signaling pathways.

Supplementary Table S1. Primers used in this study.

	Forward primer (5'->3')	Reverse primer (5'->3')
**Map-based cloning**		
RM14638	CCTGTCCCACCTCGATTTGC	CAGATCGATCCCGTGCTACTGC
RM14374	CATGAGACAGCGAAGAACAATGG	CCGGTACTGTACTGAGACTGAGAGG
RM14395	AATATCCGCAGCCGAAACATAGC	ACCGACCAAACCAACACAATCG
S3014.8	GCCACCATTTTACCTTTTTCC	TGGAAGCTGGGGGTAGTAAA
S3015.2	TGTATCCCATGACAGCAAGC	AGCTCGGAATTTCCTGTTGA
SSR-5	ACCCTTCTGTGGTTGTGTCC	GACATGAATATGTTCCTCCTGCT
RM14423	AGTCAGTCAGTCCAATTCAGTCACG	AGGACGACGACGAGTGTAACTGC
RM14430	AAGCAACGCAAGAACCCTTGG	ATGGCCGTCCAATAAACCAACC
RM5474	AAAGTGTTGGTGAGCATAGC	TTTGTGTTTGGAGAGACGAG
RM14443	GACCGCCGCTCTGATATAAAGG	TAACACCCAGTACGTCGTCAAGG
RM5474	AAAGTGTTGGTGAGCATAGC	TTTGTGTTTGGAGAGACGAG
**Quantitative real-time PCR (qPCR)**		
SPL3	TATGGGAATGCTGGCAGAA	CTGGGACTCCTGGTGAGAAG
OsABI1	AACGAGAGGCTGCACTGA	CTAGTAGCTTCCTGCTCTG
OsABI3	CTGCAGGAGGGTGATTTCAT	CTCACCTTCACACCACGTATC
OsABI4	ACCCCTGGTTCGATCTCTTC	AGCTGGAACGCCAAGCTAAG
OsABI5	CGAAGCTGAACTGAACTATC	CTGGCTGCCACCCCTATTTG
OsAREB1	CAGAGGCGGATGATCAAGAA	CCTCAGCTTCCAACTCCATTA
OsbZIP23	GAAGGTTGTCGAGAGAAGACAG	TGCTACCTCAGCTTCCAATTC
OsDSG1	AGCTGAACCTTCTGGAGATCA	CTGCAGAGATTCTTCCAAAGC
OsDSR1	GCACATACCGAGAACACCTTT	GTACGCCTCTCCAAACACAAT
OsRePRP2.1	CCAAAACCAACGCTGCAA	GGTCAGCTTTGGGGCTAGG
OsSAPK6	TGGCAGATGTATGGTCTTGTG	TGATACGATTCTCCCGATTGT
OsSAPK8	GAATATGCCTCTGGTGGTGAG	CGAGATAAGCTGCTGGAAGAA

OsSAPK9	GAGGCTCGTTTCTTCTTCCA	GCAGAGTGTTCTCCAGCTTTA
OsACS1	ACCAAGATGTCCAGCTTCGG	GAGGAGGTACTGCGTCTGGG
OsACS2	GGAATAAAGCTGCTGCCGAT	TGAGCCTGAAGTCGTTGAAGC
OsEIN2	GTTGTCAGCTTCGGTGTCTG	CCTGAAGACGGTTGAGAACA
OsEIN3	ATCTTCCCGGCAACCTACAA	CATGATCGTGGCATTGTCGT
OsNAP	AACCATTTCATCGCGAACAAC	CAGTGACGATCCCTGCAAGG
NYC1	GAATCCGTAATTGGGCTGAA	CTGGAAGAGGTCCACCTGAG
SGR	AGGGGTGGTACAACAAGCTG	GCTCCTTGCGGAAGATGTAG
OsUBQ5	ACCACTTCGACCGCCACTACT	ACGCCTAAGCCTGCTGGTT
